# Synthetic Biohybrids of Red Blood Cells and Cascaded‐Enzymes@ Metal–Organic Frameworks for Hyperuricemia Treatment

**DOI:** 10.1002/advs.202305126

**Published:** 2023-12-06

**Authors:** Zeyu Li, Liecong Xue, Junxian Yang, Stefan Wuttke, Peiying He, Chuanyi Lei, Haowei Yang, Liang Zhou, Jiangfan Cao, Anna Sinelshchikova, Guansheng Zheng, Jimin Guo, Jiangguo Lin, Qi Lei, C. Jeffrey Brinker, Kaisheng Liu, Wei Zhu

**Affiliations:** ^1^ MOE International Joint Research Laboratory on Synthetic Biology and Medicines School of Biology and Biological Engineering South China University of Technology Guangzhou 510006 P. R. China; ^2^ Medical Research Institute Guangdong Provincial People's Hospital (Guangdong Academy of Medical Sciences) Southern Medical University Guangzhou 510000 P. R. China; ^3^ BCMaterials Basque Center for Materials UPV/EHU Science Park Leioa 48940 Spain; ^4^ IKERBASQUE Basque Foundation for Science Bilbao 48009 Spain; ^5^ China National Tobacco Corporation No.55 South Yuetan Boulevard Xicheng District Beijing 100045 P. R. China; ^6^ College of Materials Sciences and Engineering Beijing University of Chemical Technology Beijing 100029 P. R. China; ^7^ The Second Affiliated Hospital State Key Laboratory of Respiratory Disease Guangdong Provincial Key Laboratory of Allergy and Clinical Immunology Guangzhou Medical University Guangzhou 510260 P.R. China; ^8^ Center for Micro‐Engineered Materials and the Department of Chemical and Biological Engineering The University of New Mexico Albuquerque NM 87131 USA; ^9^ Guangdong Provincial Clinical Research Center for Geriatrics Shenzhen Clinical Research Center for Geriatrics, Shenzhen People's Hospital (The Second Clinical Medical College, Jinan University; The First Affiliated Hospital, Southern University of Science and Technology) Shenzhen 518020 P. R. China

**Keywords:** biohybrid materials, red blood cells, Metal‐organic Frameworks, enzyme, hyperuricemia

## Abstract

Hyperuricemia, caused by an imbalance between the rates of production and excretion of uric acid (UA), may greatly increase the mortality rates in patients with cardiovascular and cerebrovascular diseases. Herein, for fast‐acting and long‐lasting hyperuricemia treatment, armored red blood cell (RBC) biohybrids, integrated RBCs with proximal, cascaded‐enzymes of urate oxidase (UOX) and catalase (CAT) encapsulated within ZIF‐8 framework‐based nanoparticles, have been fabricated based on a super‐assembly approach. Each component is crucial for hyperuricemia treatment: 1) RBCs significantly increase the circulation time of nanoparticles; 2) ZIF‐8 nanoparticles‐based superstructure greatly enhances RBCs resistance against external stressors while preserving native RBC properties (such as oxygen carrying capability); 3) the ZIF‐8 scaffold protects the encapsulated enzymes from enzymatic degradation; 4) no physical barrier exists for urate diffusion, and thus allow fast degradation of UA in blood and neutralizes the toxic by‐product H_2_O_2_. In vivo results demonstrate that the biohybrids can effectively normalize the UA level of an acute hyperuricemia mouse model within 2 h and possess a longer elimination half‐life (49.7 ± 4.9 h). They anticipate that their simple and general method that combines functional nanomaterials with living cell carriers will be a starting point for the development of innovative drug delivery systems.

## Graphical abstract

Armored red blood cell (RBC) biohybrids, integrated RBCs with proximal, cascaded‐enzymes of urate oxidase and catalase encapsulated within ZIF‐8 framework‐based nanoparticles, have been fabricated for fast‐acting and long‐lasting urate‐lowering therapy. RBC biohybrids could normalize the UA level of acute hyperuricemia model mice within 2 h, and possessed an elimination half‐life of 49.7 ± 4.9 h, which is over thrice longer than that of free UOX, representing an innovative drug carrier for hyperuricemia treatment.

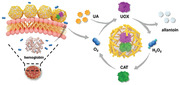

## 1 Introduction

Hyperuricemia, characterized by the excess of blood uric acid (UA) and the supersaturation of extracellular urates, is caused by purine metabolic disorder or impaired UA excretion.^[^ ^1^ ^]^ Longstanding hyperuricemia may lead to gout, and increase mortality rates in patients with cardiovascular and cerebrovascular diseases, metabolic syndrome, and kidney disease.^[^ ^2^, ^3^ ^]^ So far the mainstream therapeutic strategies focus on balancing the production and excretion of UA, for example, by inhibiting uric acid production through using xanthine oxidase inhibitors (e.g., allopurinol and febuxostat), and promoting uric acid excretion by employing renal tubular reabsorption blockers (e.g., benzbromarone and probenecid).^[^ ^4^, ^5^, ^6^ ^]^ Nevertheless, both approaches suffer from inevitable side effects involving adverse drug reactions, suboptimal antihyperuricemic efficacy, and cumulative liver toxicity.^[^ ^7^ ^]^ Alternatively, a burgeoning approach to manage hyperuricemia is the administration of urate oxidase (UOX) extracted from various sources or expressed in recombinant organisms, which catalyzes the oxidation of uric acid to allantoin, a more soluble product that can be readily excreted in the urine.^[^ ^8^, ^9^ ^]^ Despite its accelerated metabolization of UA, the clinical application of UOX is limited by the inherent antigenicity, immunogenicity, insufficient enzymatic activity, poor stability, low bioavailability, and short circulation half‐life.^[^ ^10^, ^11^ ^]^ Currently, numerous efforts have been made to overcome the above limitations including the PEGylation form of recombinant mammalian uricase to lower the immunogenicity,^[^ ^12^, ^13^, ^14^ ^]^ the micro/nano‐encapsulation form using liposomes,^[^ ^15^, ^16^ ^]^ microdroplets,^[^ ^17^ ^]^ amphoteric polymers,^[^ ^18^ ^]^ and serum albumin‐based hydrogels^[^ ^19^ ^]^ to conquer the challenges of enzymatic degradation and increase circulation time, and the further integration of catalase (CAT)‐like enzyme to both accelerate the UOX‐mediated UA degradation and enhance the enzymatic activity.^[^ ^20^, ^21^ ^]^ Nevertheless, the sustained UA degradation effects are still subject to the rapid system clearance of the totally foreign UOXs as well as their derived structures after intravenous infusion. Moreover, the efficacy of UOX‐based hyperuricemia treatment is constrained by multiple factors including catalytic activity (molecular oxygen participated) and accessibility of enzyme, byproduct (H_2_O_2_) removal rate, circulation time, and stability in the body to achieve the long‐term effect.^[^ ^22^ ^]^ These challenges lead to a need for a multifunctional integrated drug carrier that can comprehensively address these issues.

**Figure 1:**
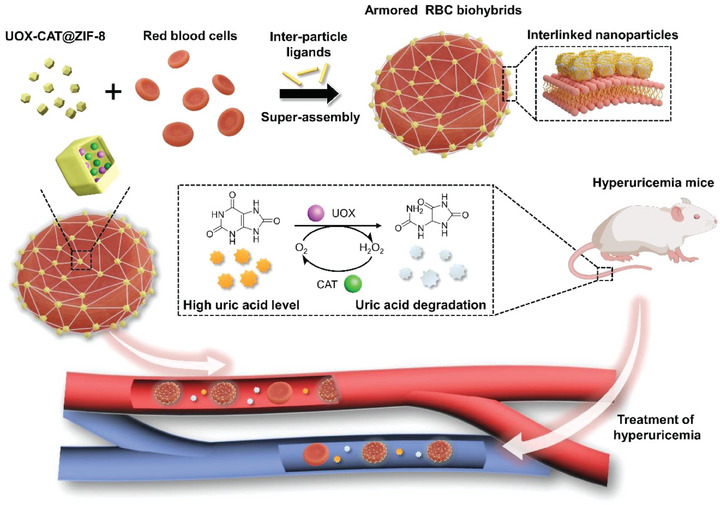
Schematic illustration of super‐assembly of cascaded‐enzymes@MOF nanoparticles‐based building blocks on the RBC surface to form armored RBC biohybrids for hyperuricemia treatment.

Red blood cells (RBCs), the most abundant cellular component in blood with about 120‐day circulation time, are responsible for transporting oxygen from the lungs to various organs and tissues.^[^ ^23^ ^]^ Recent reports have demonstrated that surface adsorption of nanoparticles on RBCs via a bioaugmentation strategy can significantly extend the circulation time of nanoparticles and regulate their in vivo distribution.^[^ ^24^, ^25^ ^]^ In our previous work, metal‐organic frameworks (MOFs) serving as nano‐building blocks and polyphenols acting as interparticle ligands have been employed and super‐assembled to construct armored RBCs, endowing erythrocyte with enhanced resistance against external stressors while preserving the original properties (such as oxygen carrier capability and good *ex ovo*/in vivo circulation property).^[^ ^26^, ^27^, ^28^ ^]^ Herein, along with the concept of bioaugmentation of RBC via modular hybridization of nanomaterials, a functional building block, cascaded enzymes (UOX‐CAT) encapsulated in ZIF‐8 (ZIF: Zeolitic imidazolate framework) NPs, have been fast super‐assembled to the surface of RBC, obtaining armored RBC biohybrids for fast‐acting and long‐lasting urate‐lowering therapy (**Figure** **1**). When intravenously administering the armored RBC biohybrids, small UA molecules in blood were accessible to the exoerythrocytic UOX and underwent efficient degradation in the UOX‐CAT cascaded catalytic reaction, and pericellular hyperoxia of RBCs would further accelerate the UA degradation rates for fast‐acting catalysis. Meanwhile, benefiting from the encapsulation in ZIF‐8 and the hitchhiking effect on RBCs, the encapsulated UOX would circumvent rapid enzymic degradation and systemic clearance in vivo, thus efficiently maintaining their intrinsic enzymatic activity for long‐lasting catalysis. Surprisingly, in vivo results demonstrated that the obtained RBC biohybrids could normalize the UA level of acute hyperuricemia model mice within 2 h, and possessed an elimination half‐life of 49.7 ± 4.9 h, which is over thrice longer than that of free UOX (14.2 ± 1.7 h). The synthesis of armored RBC biohybrids is straightforward, fast and reliable and hence, represents an innovative drug carrier for hyperuricemia treatment.

## 2 Results and Discussion

### 2.1 Construction of Cascaded‐Enzymes@Metal–Organic Framework

**Figure 2:**
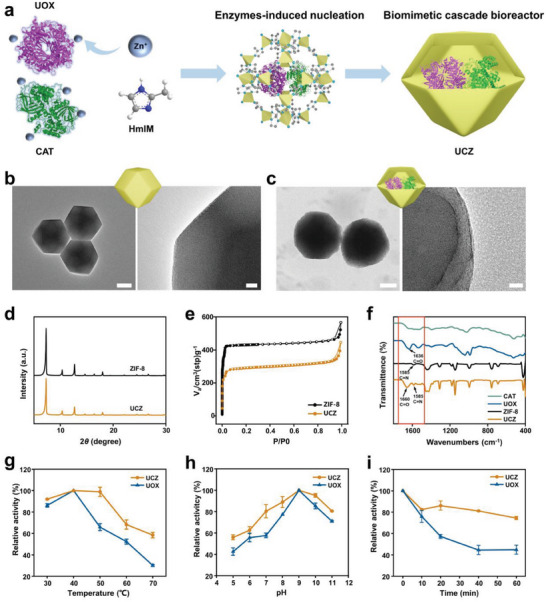
A) Schematic illustration of UCZ synthesis. B) TEM images of ZIF‐8. Scale bars, 100 nm (left), 10 nm (right). C) TEM images of UCZ. Scale bars, 100 nm (left), 10 nm (right). D) XRD patterns of ZIF‐8 and UCZ. E) BET profiles of ZIF‐8 and UCZ. F) FT‐IR spectra of ZIF‐8, CAT, UOX, and UCZ. Relative catalytic activity of UOX and UCZ on uric acid degradation under different interventions of temperature G), pH H) and trypsin digestion I).

In a typical synthesis, ZIF‐8, a frequently used enzyme immobilization carrier, was utilized for dual‐enzyme encapsulation, because of the mild synthesis condition in aqueous phase that can well maintain the enzyme activities.^[^ ^29^, ^30^, ^31^ ^]^ UOX‐CAT@ZIF‐8 (denoted as UCZ) was synthesized by a one‐pot method, in which UOX and CAT were premixed with a 2‐methylimidazole solution and then added to a zinc nitrate solution (**Figure** **2**a).^[^ ^32^ ^]^ Specifically, UCZ acts as a miniature bioreactor where the dual enzymes UOX and CAT are confined, leading to an accelerated cascade reaction rate due to the proximity effect.^[^ ^33^ ^]^ During the degradation of uric acid (UA) mediated by UOX, the decomposition of the toxic byproduct hydrogen peroxide (H_2_O_2_) could be facilitated by the nearby CAT.

To visualize the physical structure of ZIF‐8 and UCZ, the synthesized nanoparticles were examined under transmission electron microscope (TEM) and scanning electron microscope (SEM). Figure 2b shows that ZIF‐8 has a standard rhombic dodecahedral structure, while enzyme encapsulated UCZ have slightly larger‐size and spherical‐like morphology (Figure 2c and Figure S1, Supporting Information). The XRD pattern of UCZ was consistent with that of ZIF‐8, indicating that the crystal structure and crystallinity of ZIF‐8 were well maintained (Figure 2d). After being preserved in water solution for 2 weeks, the XRD pattern of UCZ remained unchanged, indicating the long‐term stability in aqueous phase (Figure S2, Supporting Information). Moreover, after exposure to PBS and plasma for 1 week, the basic rhombohedral shape of ZIF‐8 nanoparticles still remained highly distinct, indicating the long‐term stability of ZIF‐8 in physiological environments (Figure S3, Supporting Information). Moreover, nitrogen adsorption/desorption isotherms of both nanoparticles were type I (Figure 2e), confirming the presence of microporous structures. The specific surface area of ZIF‐8 was 1899 m^2^/g, while that of UCZ was reduced to 1310 m^2^/g owing to the internal loading of UOX and CAT. The loading of both enzymes in UCZ was further proved by Fourier transform infrared spectroscopy in Figure 2f. The spectral band of ZIF‐8 at 1585 cm^−1^ primarily originates from C = N stretching vibrations of the assembled methylimidazole. The FTIR spectrum of UCZ also recorded this characteristic peak, and an additional spectral band appeared at 1660 cm^−1^, which could be attributed to the amide I band of C = O stretching of UOX and CAT, confirming the successful encapsulation.

To achieve the best cascade catalytic activity in UCZ system, the loading ratio of two enzymes (UOX and CAT) has been optimized. As shown in Figure S4 (Supporting Information), the by‐product H_2_O_2_ could be completely degraded when the molar ratio of UOX to CAT was ≤4:1, while the fastest UA degradation rate was observed at the ratio of 4:1. Therefore, for the follow‐up experiments the ratio of UOX to CAT was chosen to be 4:1 for cascaded enzyme encapsulation. To determine the actual molar ratio, UOX and CAT were fluorescently labeled with Cyanine7 NHS ester (Cy7‐NHS) and Fluorescein isothiocyanate (FITC), respectively, and then the standard curve was measured by a fluorescence spectrophotometer. Based on the fluorescence, the enzyme loading of 13.91% for UOX and 7.69% for CAT, has been measured, corresponding to an actual molar ratio of ≈7:2 (Figures S5 and S6, Supporting Information).

Improving the stability of exogenous enzymes is crucial for their in vivo applications.^[^ ^34^, ^35^ ^]^ Therefore, we further inspected the effects of several environmental conditions, such as pH, temperature, and trypsin treatment on enzyme activities. As displayed in Figure 2g, within the measured temperature range, the residual activity of UCZ was higher than that of free UOX, and its enzyme activity was approximately twice that of UOX at 70 °C. Moreover, over the wide tested pH range, the residual activity of UCZ was also higher than that of free UOX (Figure 2h). For trypsin treatment, UOX had only 57% enzyme activity after 20 min, whereas the activity of UCZ decreased more slowly and remained above 80% within 40 min (Figure 2i). The above results revealed that with the encapsulation of UOX in ZIF‐8‐based framework, UCZ exhibited dramatically enhanced resistance to pH, high temperature, and protease degradation compared to free uricase.

### 2.2 Construction of Armored RBC Biohybrids

**Figure 3:**
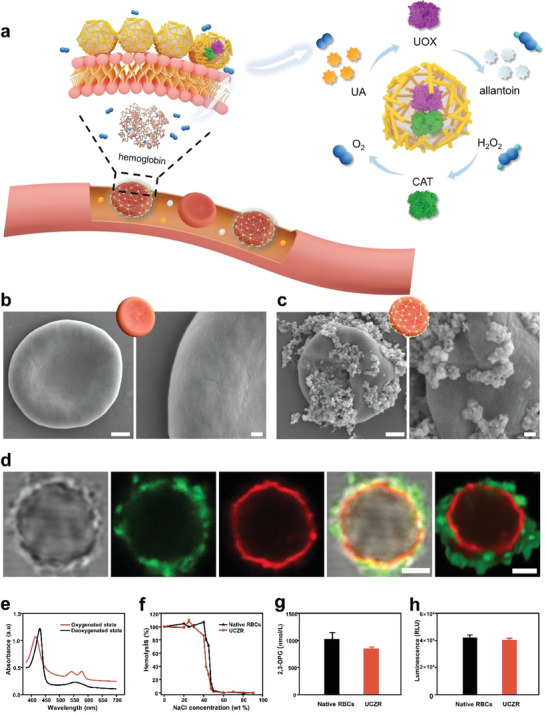
A) Schematic illustration of the activation of a dual‐enzyme biocatalytic cascade encapsulated within ZIF‐8. Left, the oxygen molecules carried by hemoglobin help facilitate uric acid degradation. Right, enzymatic cascade driven by the two biocatalysts. B) SEM image of native RBCs (left) and its magnified image (right). Scale bars 1 µm (left), 200 nm (right). C) SEM image of UCZR (left) and its magnified image (right). Scale bars 1 µm (left), 200 nm (right). D) Images of UCZR were obtained by labeling UOX and RBC with FITC and Dil, respectively (bright field, FITC, Dil, combined image, and Z‐scan image, from left to right). Scale bars, 2 µm. E) UV–vis spectra of the oxygenated and deoxygenated states of UCZR. F) Osmotic fragility curves of native RBCs and UCZR. 2,3‐DPG G) and ATP H) contents of native RBCs and UCZR.

By employing UCZ as nano‐building blocks and tannic acid as interparticle ligands, the dual‐enzymes@ZIF‐8 nanoparticles could be fast assembled around RBC surface to form nanoparticles‐based exoskeletons. The assembly process consists of two steps: 1) Under pH 5.0 condition, hybrid nanoparticles are incubated with RBCs for 30 s, enabling the nanoparticles to attach and accumulate on the cell surface driven by hydrogen bond interactions between the organic ligands of the particles and cell surface proteins; 2) Adding tannic acid ligands, the interlocking between particles is realized through strong multivalent coordination between metal ions and polyphenols.^[^ ^36^, ^37^ ^]^ All the assembly process could be finished within 1 min, ultimately forming the desired armored RBC biohybrids, designated as UOX‐CAT@ZIF‐8‐RBC (UCZR). The SEM images of RBCs before and after nanoparticles super‐assembly are displayed in **Figure** **3**b,c. The native RBCs exhibited distinct biconcave structures, while significant dense particle accumulation was observed on the surfaces of UCZR. Energy‐dispersive X‐ray spectroscopy (EDX) analysis revealed a striking increase in Zn content for the case of UCZR, suggesting the successful assembly of nanoparticles around RBC surface (Figure S8, Supporting Information). After labeling CAT and RBC membranes with FITC and DiIC_18_(3) (Dil), respectively, uniform red fluorescence was observed on the RBC membrane under a laser confocal scanning microscope (CLSM). The even distribution of green fluorescent UCZ nanoparticles was visible outside of the red fluorescence. This observation verified that RBCs were encapsulated by the UCZ‐based exoskeleton (Figure 3d). Notably, the structure of RBC biohybrids remained stable after prolonged storage. Even after 7 days of storage at room temperature, the biohybrids on the surface of RBCs protected erythrocytes from hemolysis (Figure S9, Supporting Information), indicating that the formed exoskeleton is non‐toxic to erythrocytes.

Erythrocytes play a crucial role in oxygen transportation of the physiological activities of human beings.^[^ ^38^ ^]^ By assembling an UCZ‐based exoskeleton on the RBC surface, the conversion of UA into allantoin can benefit from the abundant oxygen of the RBC biohybrids, whose physiological function is as usual as the native RBCs. To evaluate the oxygen‐carrying capacity of UCZR, UV‐vis spectroscopy was utilized to reveal the reversible shifts in maximum absorption peaks under oxygenated and deoxygenated states (Figure 3e). The characteristic absorption peaks of native RBCs and UCZR in the oxygenated state are 415 nm. The absorption peak red‐shifted to 430 nm after nitrogen injection for 2 h and the addition of the reducing agent sodium dithionite (Na_2_S_2_O_4_), confirming the transition of UCZR to the deoxygenated state. Moreover, the formed RBC superstructures could regain oxygen after being exposed their deoxygenated state to the atmosphere (Figure S10, Supporting Information). The process of binding and releasing oxygen was repeatable, indicating that the UCZR retained the oxygen‐carrying and releasing capacity of native RBCs. Furthermore, regarding to the deformability of the formed biohybrids, microfluidic blood vasculature capillary model has been constructed for investigation. As shown in Figure S11 (Supporting Information), ZIF‐8‐RBC biohybrids can successfully traverse channels with a width and height of 5 micrometers, demonstrating their normal deformability. Subsequently, we collected ZIF‐8‐RBC biohybrids before and after passing through the channels and observed the FITC‐labeled nanoparticles on them. We found that the fluorescence intensity on ZIF‐8‐RBC biohybrids passing through the chip channels exhibited only a slight reduction, indicating that the majority of nanoparticles could be well retained (Figure S12, Supporting Information).

Moreover, luminol‐based chemiluminescence was applied to detect the presence of hemoglobin in UCZR and its iron catalytic activity (Figure S13, Supporting Information). The ferrous iron in hemoglobin accelerates the reaction of luminol with the peroxide produced by perborate, generating blue light.^[^ ^39^ ^]^ When luminol‐perborate was added to the solution, chemiluminescence was observed from both native RBCs and UCZR, suggesting that the incorporation of the nanoparticles‐based exoskeleton does not interfere with the iron‐mediated catalytic activity of hemoglobin. The inherent pores ensure full accessibility of small molecules, such as luminol and peroxides, to the RBCs and facilitate their traversal across the RBC membrane.

Next, the osmotic pressure tolerance of the armored RBC biohybrids was examined. As shown in Figure 3f, the osmotic fragility curves of native RBCs and UCZR were strikingly similar. Both native RBCs and UCZR began to rupture at NaCl concentration below 0.60% (w/v). When the concentration was as low as 0.40%, native RBCs absolutely ruptured, while the armored RBC biohybrids did not break completely until the concentration was 0.30%. This resistance to the increased osmotic pressure of UCZR may be attributed to the physical confinement from MOF nanoparticles‐based exoskeleton for RBC structure augmentation and the inherent porosity of MOFs to slow down the diffusion of ions from external medium.^[^ ^40^ ^]^ Furthermore, the levels of 2,3‐diphosphoglycerate (2,3‐DPG) and adenosine triphosphate (ATP) in the UCZR were tested and found to be slightly lower than those in native RBCs, but without notable changes (Figure 3g,h). Besides, according to the results of SDS‐PAGE, the UCZR still retained all the membrane protein components of native RBCs, indicating no significant damage to erythrocytes (Figure S15, Supporting Information). Note that, as shown in Figure S16 (Supporting Information), there is no significant loss of CD47 protein in UCZR, which is highly favorable for the circulation of UCZR.

### 2.3 Mechanical Property Study

**Figure 4:**
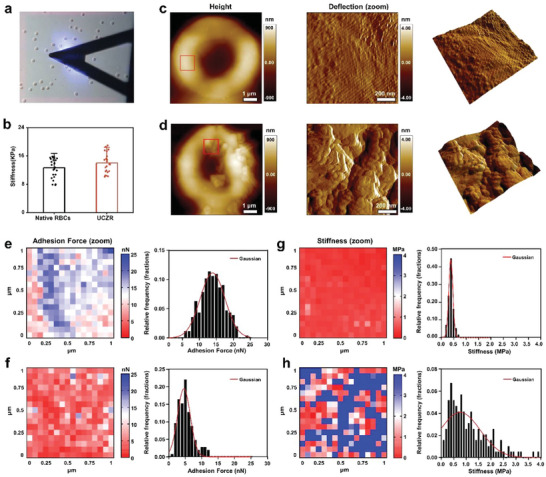
A) Image of scanning RBC by AFM. B) Young's modulus of native RBCs and UCZR. Whole‐cell images and zoomed‐in images of native RBCs C) and UCZR D) by AFM. Heatmaps and relative frequency histograms of adhesion forces of zoomed‐in area in native RBCs E) and UCZR F). Heatmaps and relative frequency histograms of Young's modulus of zoomed‐in area in native RBCs G) and UCZR H).

Erythrocytes are widely used for drug delivery in vivo due to their unique biocompatibility and long‐circulation time, and their outstanding deformability allows them to effortlessly pass through microvasculature narrower than themselves.^[^ ^41^, ^42^ ^]^ The deformability of RBCs refers to the ability to return to the original biconcave disc shape after deformation, which is related to the elasticity, stiffness, and hardness of the RBC membrane.^[^ ^43^ ^]^ RBC deformability tremendously affects its transportation to various tissues and organs.^[^ ^44^ ^]^ To this end, an atomic force microscope (AFM) was utilized to observe the changes in the morphology and mechanical properties of RBCs before and after assembly of MOF nanoparticles. As a classic high‐resolution biological surface morphologic imaging tool, AFM can reveal the surface morphologic changes of RBCs (**Figure** **4**a).^[^ ^45^, ^46^ ^]^ Upon testing Young's modulus on 25 native RBCs and UCZR, it was found that Young's modulus of the UCZR group was 14.04 ± 2.86 MPa, which was slight higher than native RBCs of 12.66 ± 2.67 MPa (Figure 4b). The typical biconcave structure was observed for both samples. The regular nano‐network structure of the surface membrane protein can be seen by zooming in on the 1 µm^2^ area of the cell surface, the native RBCs surface is smooth while the surface of UCZR is very rough, displaying noticeable particles in the enlarged part (Figure 4c,d). In addition, the force‐distance curves generated by the cantilever tip in the vertical direction can provide information about the elasticity, hardness, and adhesion force of the RBC surface. Due to the existence of MOF nanoparticles‐based exoskeleton, the cell adhesion after loading nanoparticles was 13.89 ± 0.22 nN, which is larger than 5.17 ± 0.15 nN that of native cells (Figure 4e,f). Moreover, the test of Young's modulus of the magnified 1 µm^2^ part revealed increased surface hardness of the RBC loaded with nanoparticles. The Young's modulus of the UCZR was 5.43 ± 0.71 MPa, which was higher than 0.39 ± 0.01 MPa for untreated RBCs (Figure 4g,h).

### 2.4 Cytotoxicity Assay

Before characterizing the in vivo circulation properties of cascaded‐enzymes@ZIF‐8 loaded RBC biohybrids, we first assessed their cytotoxicity. To this end, the cytotoxicity of different concentrations of UCZ and UCZR on Human Skin Fibroblast (HSF) cells was conducted. As shown in Figure S17 (Supporting Information), both samples demonstrated remarkably low cytotoxicity even at concentrations up to 200 µg/mL, implying the good biocompatibility. Moreover, the hemolysis assay was employed to assess the cell compatibility of UCZR, and no obvious hemolysis was observed at the measured concentration (Figure S18, Supporting Information). Furthermore, the cytotoxicity in the presence of UA was investigated. In the presence of UA, compared to uricase‐treated 4T1 cells that displayed lower cell viability possibly because of uricase‐catalyzed UA producing toxic H_2_O_2_, the survival rate of cells treated with UCZ and UCZR was strikingly increased (Figure S19, Supporting Information). The results were also verified by confocal laser scanning microscopy imaging. It was observed that the application of uricase resulted in massive cell death in the presence of UA, whereas almost no dead cells were found in the cells treated with UCZ and UCZR (Figure S20, Supporting Information). We hypothesized that the cytotoxicity resulted from the production of reactive oxygen species (ROS), such as H_2_O_2_. To verify the above speculation, we used 2′,7′‐dichlorofluorescein diacetate (DCFH‐DA) to monitor the intracellular ROS levels in 4T1 cells. As revealed in Figure S21 (Supporting Information), in the presence of UA, substantial green fluorescence can be observed in UOX‐treated cells, proving the production of a vast quantity of ROS. However, cells treated with UCZR show negligible green fluorescence, indicating that the establishment of the dual‐enzyme cascade system can effectively remove ROS, that is, the oxidation product H_2_O_2_ of UA can be decomposed by CAT in time. Our findings strongly demonstrate that the designed UCZR has minimal toxic side effects for the treatment of hyperuricemia. Taken together, by constructing a dual‐enzyme cascade system and using the RBCs as carriers, the safety of treatment can be significantly improved.

### 2.5 In Vivo Imaging

**Figure 5:**
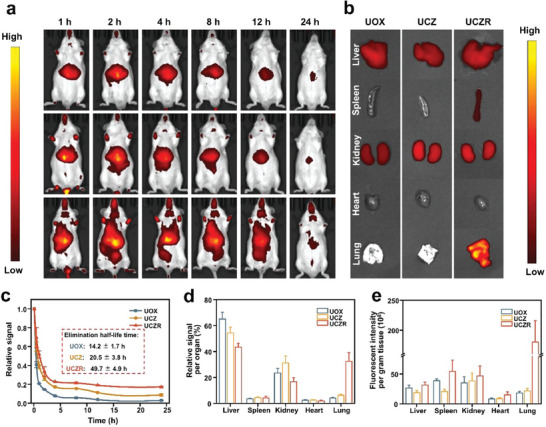
A) In vivo fluorescence images of whole mice taken at 1, 2, 4, 8, 12, and 24 h after being intravenously injected with Cy7‐labeled UOX (top), UCZ (middle), and UCZR (bottom), respectively. B) Fluorescence images of major organs (liver, spleen, kidney, heart, and lung from top to bottom) after intravenous injection of UOX, UCZ, and UCZR for 24 h. C) Pharmacokinetic curves of UOX, UCZ, and UCZR. The insert table is the associated elimination half‐life. Fluorescence intensity per gram of tissue D) and relative fluorescence signal per organ E) after intravenous injection of UOX, UCZ, and UCZR for 24 h.

The hitchhiking effect of UCZ on the RBC surface is expected to enhance their circulation half‐life. To explore the pharmacokinetic and biodistribution behaviors of UCZR, KM mice were injected with Cy7‐labeled free UOX, UCZ, and UCZR at a dosage of 240 µg nanoparticles per mouse via tail vein injection. The UCZR were made using syngeneic RBCs, which eliminates the rejection reactions caused by blood type incompatibility. To investigate the circulation half‐life, at various time points post‐injection (**Figure** **5**a), blood was collected from the tail of the mice to assess the concentrations of free UOX, UCZ, and UCZR. At 12 h and 24 h post‐injection, the total retention rate of the UCZR in mice blood was 19.1% and 17.2%, respectively, compared to 11.3% and 8.8% of the control nanoparticles (Figure 5c). The semilog plot of retention‐circulation time reveals a bi‐exponential decrease in particle concentration over time, confirming that UCZ and UCZR circulation followed a two‐compartment pharmacokinetic model (Figure S22, Supporting Information).^[^ ^47^ ^]^ According to the two‐compartment pharmacokinetic simulation analysis, the elimination half‐life of nanoparticles and UCZR were 20.5 ± 3.8 h and 49.7 ± 4.9 h, respectively. Compared with nanoparticles, the retention of UCZR in blood circulation was significantly enhanced. The result implies that attaching nanoparticles to the RBC surface can indeed prolong the circulation of nanoparticles, where the vascular mobility, circulation, and flexibility of RBCs could aid the adherent nanoparticles to avoid clearance by the rapid reticuloendothelial system (RES).^[^ ^48^, ^49^ ^]^

Additionally, to investigate the distribution within relevant organs, mice were euthanized at 24 h after injection, and their liver, spleen, kidney, heart, and lungs were collected for fluorescence analysis (Figure 5b). The UCZR displayed a prominent fluorescence signal in the lungs, besides the majority of fluorescence signals being found in the two primary filtration organs (liver and spleen) (Figure 5e,f). This result may be attributed to the fact that UCZ nanoparticles on the surface of the biohybrids accumulate in the lungs after being squeezed off when passing through the narrow capillaries in the pulmonary vasculature system. Shear forces generated during the substantial volume of cardiac blood output facilitate the transfer of nanoparticles from the surface of RBCs to the endothelial cells of lung capillaries.^[^ ^50^ ^]^ Note that the in vivo stability or separation behavior of RBC biohybrids is currently difficult to characterize. To sum up, our created RBC biohybrids exhibit enhanced in vivo retention times compared to nanoparticles.

### 2.6 Hyperuricemia Treatment

**Figure 6:**
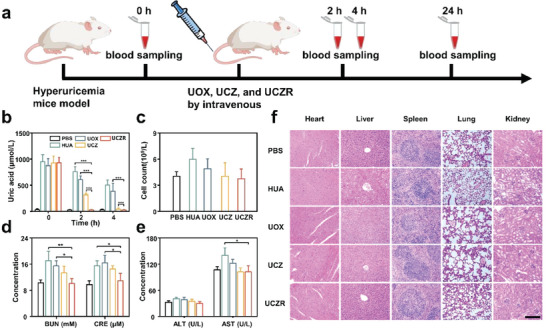
A) Schematic illustration of hyperuricemia mice model establishment and treatment process. B) BUA concentrations in hyperuricemia model mice treated with different samples (mean± standard deviation, n = 3, two‐tailed t‐test). C) WBC counts in hyperuricemia mice treated with different samples. D) ALT and AST levels in hyperuricemia model mice treated with different samples (mean± standard deviation, n = 3, two‐tailed t‐test). E) BUN and CRE levels in hyperuricemia model mice treated with different samples (mean± standard deviation, n = 3, two‐tailed t‐test). F) Histological analysis of explanted organs (liver, spleen, kidney, heart, and lung) using hematoxylin and eosin staining. (*P <0.05, **P < 0.01 and ***P <0.001).

After verifying the ability of UCZR to degrade UA in vitro, a mouse model of hyperuricemia was established to further examine the capability of UCZR in vivo. As depicted in **Figure** **6**a, the hyperuricemia mouse model was induced by subcutaneous injections with oxonic acid potassium at a dosage of 100 mg kg^−1^ and intraperitoneal injections of hypoxanthine at a dosage of 200 mg kg^−1^ into the KM mice.^[^ ^51^ ^]^ After that, we randomly divided the mice into four groups, which were intravenously injected with PBS, UOX, UCZ, and UCZR at the same enzyme dose of 25 U kg^−1^. The plasma UA concentration was detected at regular intervals, with healthy mice used as the control group. As illustrated in Figure 6b, with the change of time, the UA levels of UCZR‐treated mice rapidly dropped to the same level as those of healthy mice within 2 h, while those of PBS‐treated mice remained at high levels. As well, mice treated with UOX and UCZ exhibited higher levels than those UCZR‐treated mice. This result indicated that the designed RBC biohybrids could efficiently treat hyperuricemia, demonstrating the potential for clinical therapy of hyperuricemia.

The in vivo toxicity of UCZR was comprehensively assessed based on blood biochemistry and histopathology, as H_2_O_2_ was produced during UA degradation and therefore damaged normal cells.^[^ ^52^ ^]^ Abnormal apoptosis induced by H_2_O_2_ may result in the proliferation of immune cells, such as white blood cells (WBCs). As presented in Figure 6c, the WBC level of UOX‐treated mice (UOX = 4.89 ± 1.12 × 10^9^ L^−1^) was higher than UCZR‐treated group (UCZR = 3.77 ± 1.09 × 10^9^ L^−1^). Since lactate dehydrogenases (LDHs) is usually present in living cells, the detection of serum LDHs denotes abnormal cell apoptosis.^[^ ^53^ ^]^ Levels of serum LDHs also exhibited a similar phenomenon. The level of LDHs in UCZR‐treated mice was like those treated with PBS, but conspicuously lower than those in the HUA and UOX groups (Figure S23, Supporting Information). These findings demonstrated that apoptosis induced by UCZR was negligible because of its excellent H_2_O_2_ elimination ability, which was consistent with the in vitro cytotoxicity results.

Then hepatotoxicity and nephrotoxicity were evaluated by measuring post‐injection levels of alanine aminotransferase (ALT), aspartate aminotransferase (AST), blood urea nitrogen (BUN), and creatinine (CRE). As seen in Figure 6d, the levels of ALT and AST in UCZR‐treated mice were immensely lower than those of UOX‐treated mice, but equivalent to those of normal mice. In addition, BUN and CRE showed similar trends, suggesting insignificant liver and kidney damage owing to the effective elimination of UCZR to H_2_O_2_ (Figure 6e). Additionally, we analyzed plasma levels of inflammatory cytokines, such as IL‐6 and TNF‐α. As shown in Figures S24 and S25, the levels of IL‐6 and TNF‐α in UCZR‐treated mice were markedly lower than those in the model group, which was mainly because CAT could timely eliminate H_2_O_2_ produced during UA decomposition by UOX. Furthermore, hematoxylin and eosin (H&E) staining of heart, liver, spleen, lung, and kidney sections showed no striking signs of abnormal cell morphology, inflammation, or tissue structure in the UCZR group compared to the normal group (Figure 6f), while the UOX‐treated mice had severe hepatocyte injury and dilatation of glomeruli and renal tubules. This further indicated that UCZR had no significant side effects on mice. The pathological changes of the organs of mice in each group were consistent with the blood biochemical indexes, and these findings confirmed the overall biocompatibility of UCZR. Together, these in vivo study results demonstrate that UCZR has a faster UA‐lowering impact and fewer toxic side effects, with superior biosafety and non‐immunogenicity, demonstrating its potential for the treatment of hyperuricemia.

## 3 Conclusions

In summary, we utilized an artificial bioaugmentation approach to construct armored RBC biohybrids, which maintained the original properties of RBCs and further equipped them with powerful anti‐hyperuricemia ability. In combination with UOX and CAT, the dual‐enzymes@ZIF‐8 nanoparticles bound to the RBC surface could effectively degrade UA in blood via initiating a UOX‐CAT cascaded catalytic reaction augmented/enhanced by the hyperoxia microenvironment of RBCs, thus resulting in fast‐acting and long‐lasting treatment of hyperuricemia. Unlike previously reported nanomaterials‐based UOX formulations, the immobilized UOX in “armored RBC biohybrids” could circumvent rapid enzymatic degradation and systemic clearance in vivo as well as ensure UOX accessibility to UA by virtue of their hitchhiking on RBCs, thus efficiently maintaining their intrinsic enzymatic activity. Also, the fabrication method of the RBC biohybrids is simple and fast, and amenable to substitution with other functional agents. The RBC biohybrids of UCZR possessed an elimination half‐life of 49.7 ± 4.9 h, which is over twice that of nanosized UCZ (20.5 ± 3.8 h) and over three times longer than that of free UOX (14.2 ± 1.7 h). Surprisingly, the UCZR displayed a superior ability to normalize the UA level of acute hyperuricemia model mice within 2 h. Meanwhile, CAT‐mediated H_2_O_2_ elimination further avoided proliferation of WBCs, elevation of LDH levels, and pathological changes of the main organs, all demonstrating the superior biocompatibility of UCZR in vivo. All in all, our work shows the power of artificial bioaugmentation through assembling functional nanomaterials on living cell carriers and holds great promises for the development of innovative drug delivery systems.

## 4 Experimental Section

### RBC Purification

All the animal procedures complied with the guidelines of the South China University of Technology Institutional Animal Care and Use Committee. All blood samples were drawn and kept in KWS blood collection tubes (KWS Medici, Shijiazhuang, China), which have 1.5 mg of EDTA per mL of blood for anticoagulation purposes. The Ficoll‐PaqueTM PLUS density gradient centrifugation procedure was used to purify the whole blood. Then RBCs were repeatedly washed in 1X PBS (pH 7.4).

### Synthesis of Red Blood Cell Biohybrids

500 µL 1X PBS (pH 5) solution containing 400 µg/mL UCZ nanoparticles was used to suspend 5 million RBCs. After 10 s swirling and 20 s incubation, 500 µL of 32 µg/mL tannic acid in 1X PBS (pH 7.4) solution were added with 30 s vigorous mixing. After forming the red blood cell biohybrids, 1X PBS (pH 7.4) was used to rinse them off and store them.

### Tolerance against ion strength

Native RBCs and UCZR were rinsed with 1X PBS (pH 7.4) solution and then suspended in different concentrations of NaCl solution (0.9, 0.8, 0.7, 0.6, 0.5, 0.475, 0.45, 0.425, 0.4, 0.3, 0.25, 0.2, 0, w/v). Centrifuge at 200 g for 5 min after 1 h of incubation at 37 °C. The absorbance of hemoglobin in the supernatant at 540 nm was measured by a BioTek microplate (Synergy HTX) to calculate the hemolysis percentage, and draw the permeability brittleness curve.

### Confocal microscopy imaging

To verify the uniform encapsulation of UCZ nanoparticles on the surface of RBC, CAT and RBCs were fluorescently labeled with FITC and Dil. The resulting FITC labeled UCZR were rinsed with 1X PBS three times and stored in 1X PBS. Then UCZR solution was added to the confocal dish for further imaging. Confocal images were acquired with a ×100/1.4NA oil objective in sequential scanning mode using the Leica TCS SP8 confocal scanning microscope imaging system.

### In Vivo Circulation and Biodistribution Test

Six‐week‐old male KM mice were subjected to an alternating light and dark culture for 12 h at about 22 °C. Fluorescently labeled UOX, UCZ, and UCZR were administered into the mice through the tail vein, followed by in vivo imaging at various time points (1, 2, 4, 8, 12, and 24 h). 24 h later, the experimental mice were euthanized, and biological organs such as the heart, liver, spleen, lung, and kidney were collected. Their distribution in different biological tissues in vivo was analyzed by the IVIS fluorescence imaging system.

### Pharmacokinetic Tests

Six‐week‐old male KM mice were selected for alternating light and dark culture for 12 h at ≈22 °C. We randomly divided the mice into three groups. Fluorescently labeled UOX, UCZ, and UCZR were administered intravenously at a dosage of 25 U/kg. Blood samples were obtained from the tail vein of mice at different time points (0.5, 1, 2, 4, 8, 12, and 24 h) post‐administration. The changes in fluorescence over time were measured by fluorescence spectrophotometers to investigate their circulation abilities in the blood. Draw the pharmacokinetic curve based on the collected data.

## Conflict of Interest

The authors declare no conflict of interest.

## Author Contributions

L.X. and Z.L. contributed equally to this work. K.L. and W.Z. designed the project and discussed the results with all other authors. The manuscript was written through the contributions of all authors. All authors have given approval to the final version of the manuscript.

## Supporting information

Supporting Information

## Data Availability

The data that support the findings of this study are available from the corresponding author upon reasonable request.

## References

[1] J. R. Asplin , Semin. Nephrol. 1996, 16, 412. 8890397

[2] C. L. Benn , P. Dua , R. Gurrell , P. Loudon , A. Pike , R. I. Storer , C. Vangjeli , Front. Med. 2018, 5. 10.3389/fmed.2018.00160 PMC5990632 29904633

[3] T. Pascart , F. Lioté , Rheumatology 2018, 58, 27. 10.1093/rheumatology/key002 29547895

[4] A. B. Michael , S. H. Ralph , P. A. MacDonald , L. Eric , L. Christopher , J Rheumatol Suppl 2009, 36, 1273.

[5] C. M. Burns , R. L. Wortmann , Lancet 2011, 377, 165. 20719377 10.1016/S0140-6736(10)60665-4

[6] S. E. Sattui , A. L. Gaffo , Ther. Adv. Musculoskeletal Dis. 2016, 8, 145. 10.1177/1759720X16646703 PMC4959626 27493693

[7] C. L. Benn , P. Dua , R. Gurrell , P. Loudon , A. Pike , R. I. Storer , C. Vangjeli , Front Med. 2018, 5, 160. 10.3389/fmed.2018.00160 PMC5990632 29904633

[8] M. London , P. B. Hudson , Science 1957, 125, 937. 13421703 10.1126/science.125.3254.937

[9] G. Masera , M. Jankovic , M. G. Zurlo , A. Locasciulli , M. R. Rossi , C. Uderzo , M. Recchia , J. Pediatr. 1982, 100, 152. 6948943 10.1016/s0022-3476(82)80259-x

[10] N. Schlesinger , P. E. Lipsky , Semin. Arthritis Rheum. 2020, 50, S31. 32620200 10.1016/j.semarthrit.2020.04.011

[11] S. J. Kelly , M. Delnomdedieu , M. I. Oliverio , D. L. Williams , M. G. P. Saifer , M. R. Sherman , T. M. Coffman , A. G. Johnson , M. S. Hershfield , J. Am. Soc. Nephrol. 2001, 12, 1001. 11316859 10.1681/ASN.V1251001

[12] P. Caliceti , Adv Drug Deliv Rev 2003, 55, 1261. 14499706 10.1016/s0169-409x(03)00108-x

[13] Y. Yasuda , T. Fujita , Y. Takakura , M. Hashida , H. Sezaki , Chem. Pharm. Bull. 1990, 38, 2053. 10.1248/cpb.38.2053 1702692

[14] M. R. Sherman , M. G. P. Saifer , F. Perez‐Ruiz , Adv Drug Deliv Rev 2008, 60, 59. 17826865 10.1016/j.addr.2007.06.011

[15] W. Liu , J. Wu , X. Ji , Y. Ma , L. Liu , X. Zong , H. Yang , J. Dai , X. Chen , W. Xue , Theranostics 2020, 10, 6245. 32483451 10.7150/thno.45456 PMC7255035

[16] H. Xiong , Y. Zhou , Q. Zhou , D. He , X. Deng , Q. Sun , J. Zhang , Nanomed.: Nanotechnol., Biol. Med. 2016, 12, 1557.

[17] M. Zhuang , Y. Zhang , S. Zhou , Y. Zhang , K. Wang , J. Nie , J. Liu , Chem. Commun. 2019, 55, 13880. 10.1039/c9cc07037k 31675031

[18] X. Zhang , W. Chen , X. Zhu , Y. Lu , ACS Appl. Mater. Interfaces 2017, 9, 7972. 28194937 10.1021/acsami.6b16413

[19] J. Cho , S. H. Kim , B. Yang , J. M. Jung , I. Kwon , D. S. Lee , J. Controlled Release 2020, 324, 532. 10.1016/j.jconrel.2020.05.037 32454120

[20] X. Liu , Z. Zhang , Y. Zhang , Y. Guan , Z. Liu , J. Ren , X. Qu , Adv. Funct. Mater. 2016, 26, 7921.

[21] S. Jung , I. Kwon , Sci. Rep. 2017, 7, 44330. 28287162 10.1038/srep44330 PMC5347090

[22] Z. Zhang , Y. Gu , Q. Liu , C. Zheng , L. Xu , Y. An , X. Jin , Y. Liu , L. Shi , Small 2018, 14, 1801865. 10.1002/smll.201801865 30035856

[23] C. H. Villa , A. C. Anselmo , S. Mitragotri , V. Muzykantov , Adv Drug Deliv Rev 2016, 106, 88. 26941164 10.1016/j.addr.2016.02.007 PMC5424548

[24] J. S. Brenner , D. C. Pan , J. W. Myerson , O. A. Marcos‐Contreras , C. H. Villa , P. Patel , H. Hekierski , S. Chatterjee , J.‐Q. Tao , H. Parhiz , K. Bhamidipati , T. G. Uhler , E. D. Hood , R. Y. Kiseleva , V. S. Shuvaev , T. Shuvaeva , M. Khoshnejad , I. Johnston , J. V. Gregory , J. Lahann , T. Wang , E. Cantu , W. M. Armstead , S. Mitragotri , V. Muzykantov , Nat. Commun. 2018, 9, 2684. 29992966 10.1038/s41467-018-05079-7 PMC6041332

[25] J. Andreo , R. Ettlinger , O. Zaremba , Q. Peña , U. Lächelt , R. F. De Luis , R. Freund , S. Canossa , E. Ploetz , W. Zhu , C. S. Diercks , H. Gröger , S. Wuttke , J. Am. Chem. Soc. 2022, 144, 7531. 35389641 10.1021/jacs.1c11507

[26] J. Guo , Y. Yu , W. Zhu , R. E. Serda , S. Franco , L. Wang , Q. Lei , J. O. Agola , A. Noureddine , E. Ploetz , S. Wuttke , C. J. Brinker , Adv. Funct. Mater. 2021, 31, 2005935.

[27] W. Zhu , J. Guo , S. Amini , Y. Ju , J. O. Agola , A. Zimpel , J. Shang , A. Noureddine , F. Caruso , S. Wuttke , J. G. Croissant , C. J. Brinker , Adv. Mater. 2019, 31, 1900545. 10.1002/adma.201900545 31032545

[28] J. Cao , O. T. Zaremba , Q. Lei , E. Ploetz , S. Wuttke , W. Zhu , ACS Nano 2021, 15, 3900. 33656324 10.1021/acsnano.0c10144

[29] K. Liang , R. Ricco , C. M. Doherty , M. J. Styles , S. Bell , N. Kirby , S. Mudie , D. Haylock , A. J. Hill , C. J. Doonan , P. Falcaro , Nat. Commun. 2015, 6, 7240. 26041070 10.1038/ncomms8240 PMC4468859

[30] W. Liang , P. Wied , F. Carraro , C. J. Sumby , B. Nidetzky , C.‐K. Tsung , P. Falcaro , C. J. Doonan , Chem. Rev. 2021, 121, 1077. 33439632 10.1021/acs.chemrev.0c01029

[31] M. D. J. Velásquez‐Hernández , M. Linares‐Moreau , E. Astria , F. Carraro , M. Z. Alyami , N. M. Khashab , C. J. Sumby , C. J. Doonan , P. Falcaro , Coord. Chem. Rev. 2021, 429, 213651.

[32] Y. Pan , Y. Liu , G. Zeng , L. Zhao , Z. Lai , Chem. Commun. 2011, 47, 2071. 10.1039/c0cc05002d 21206942

[33] F. Li , Y. Zhang , F. Wang , J. Chen , B. Wang , N. Li , X. Lin , J. Zhuang , New J. Chem. 2022, 46, 6852.

[34] X. Wu , J. Ge , C. Yang , M. Hou , Z. Liu , Chem Commun 2015, 51, 13408. 10.1039/c5cc05136c 26214658

[35] W. Liang , H. Xu , F. Carraro , N. K. Maddigan , Q. Li , S. G. Bell , D. M. Huang , A. Tarzia , M. B. Solomon , H. Amenitsch , L. Vaccari , C. J. Sumby , P. Falcaro , C. J. Doonan , J. Am. Chem. Soc. 2019, 141, 2348. 30636404 10.1021/jacs.8b10302

[36] J. Zhou , Z. Lin , Y. Ju , M. A. Rahim , J. J. Richardson , F. Caruso , Acc. Chem. Res. 2020, 53, 1269. 32567830 10.1021/acs.accounts.0c00150

[37] X. Zhan , Z. Wen , X. Chen , Q. Lei , Y. Chen , L. Zhou , G. Zheng , F. Kong , J. Guo , Y. Duan , Y. Lai , P. Yin , C. J. Brinker , H. Chen , W. Zhu , Cell Rep. Phys. Sci. 2022, 3, 101103.

[38] X. Han , C. Wang , Z. Liu , Bioconjugate Chem. 2018, 29, 852. 10.1021/acs.bioconjchem.7b00758 29298380

[39] N. Doshi , A. S. Zahr , S. Bhaskar , J. Lahann , S. Mitragotri , Proc. Natl. Acad. Sci. USA 2009, 106, 21495. 20018694 10.1073/pnas.0907127106 PMC2799852

[40] P. Horcajada , S. Surblé , C. Serre , D.‐Y. Hong , Y.‐K. Seo , J.‐S. Chang , J.‐M. Grenèche , I. Margiolaki , G. Férey , Chem. Commun. 2007, 2820. 10.1039/b704325b 17609787

[41] C. H. Villa , D. C. Pan , S. Zaitsev , D. B. Cines , D. L. Siegel , V. R. Muzykantov , Ther. Delivery 2015, 6, 795. 10.4155/tde.15.34 PMC4712023 26228773

[42] Y. Li , F. Raza , Y. Liu , Y. Wei , R. Rong , M. Zheng , W. Yuan , J. Su , M. Qiu , Y. Li , F. Raza , Y. Liu , Y. Wei , R. Rong , M. Zheng , W. Yuan , J. Su , M. Qiu , Biomaterials 2021, 279, 121202. 34749072 10.1016/j.biomaterials.2021.121202

[43] J. Kim , H. Lee , S. Shin , Journal of Cellular Biotechnology 2015, 1, 63.

[44] C. Bernecker , M. Lima , T. Kolesnik , A. Lampl , C. Ciubotaru , R. Leita , D. Kolb , E. Fröhlich , P. Schlenke , G. A. Holzapfel , I. Dorn , D. Cojoc , Front Physiol 2022, 13. 10.3389/fphys.2022.979298 PMC9424772 36051915

[45] V. Sergunova , S. Leesment , A. Kozlov , V. Inozemtsev , P. Platitsina , S. Lyapunova , A. Onufrievich , V. Polyakov , E. Sherstyukova , Sensors 2022, 22, 2055. 35271203 10.3390/s22052055 PMC8914789

[46] E. Kozlova , A. Chernysh , E. Manchenko , V. Sergunova , V. Moroz , Scanning 2018, 2018, 1810515. 10.1155/2018/1810585 PMC6276460 30581527

[47] J. Guo , J. O. Agola , R. Serda , S. Franco , Q. Lei , L. Wang , J. Minster , J. G. Croissant , K. S. Butler , W. Zhu , C. J. Brinker , ACS Nano 2020, 14, 7847. 32391687 10.1021/acsnano.9b08714

[48] A. C. Anselmo , V. Gupta , B. J. Zern , D. Pan , M. Zakrewsky , V. Muzykantov , S. Mitragotri , ACS Nano 2013, 7, 11129. 24182189 10.1021/nn404853z PMC4128963

[49] D. C. Pan , J. W. Myerson , J. S. Brenner , P. N. Patel , A. C. Anselmo , S. Mitragotri , V. Muzykantov , Sci. Rep. 2018, 8, 1615. 29371620 10.1038/s41598-018-19897-8 PMC5785499

[50] I. V. Zelepukin , A. V. Yaremenko , V. O. Shipunova , A. V. Babenyshev , I. V. Balalaeva , P. I. Nikitin , S. M. Deyev , M. P. Nikitin , Nanoscale 2019, 11, 1636. 30644955 10.1039/c8nr07730d

[51] L. Zhang , C. Zhang , Z.‐N. Zhuang , C.‐X. Li , P. Pan , C. Zhang , X.‐Z. Zhang , Sci. China: Chem. 2021, 64, 616.

[52] J. Ming , T. Zhu , J. Li , Z. Ye , C. Shi , Z. Guo , J. Wang , X. Chen , N. Zheng , Small 2021, 17, e2103645. 34668309 10.1002/smll.202103645

[53] Z. Zhang , Y. Gu , Q. Liu , C. Zheng , L. Xu , Y. An , X. Jin , Y. Liu , L. Shi , Small 2018, 14, 1801865. 10.1002/smll.201801865 30035856

