# Supplementary material for: Synthetic Biohybrids of Red Blood Cells and Cascaded‐Enzymes@ Metal–Organic Frameworks for Hyperuricemia Treatment
Source: Adv Sci (Weinh). 2023 Dec 6;11(5):2305126. doi: 10.1002/advs.202305126 (PMC10837374; doi:10.1002/advs.202305126)
Supplement: Supplementary file 1 — Supporting Information [file ADVS-11-2305126-s001.pdf]

ADVANCED SCIENCE
Open Access

Supporting Information

for *Adv. Sci.*, DOI 10.1002/advs.202305126

Synthetic Biohybrids of Red Blood Cells and Cascaded-Enzymes@ Metal–Organic Frameworks for Hyperuricemia Treatment

Zeyu Li, Liecong Xue, Junxian Yang, Stefan Wuttke, Peiying He, Chuanyi Lei, Haowei Yang, Liang Zhou, Jiangfan Cao, Anna Sinelshchikova, Guansheng Zheng, Jimin Guo, Jiangguo Lin, Qi Lei, C. Jeffrey Brinker, Kaisheng Liu* and Wei Zhu**

Supporting Information

Synthetic Biohybrids of Red Blood Cells and Cascaded-Enzymes@ Metal-Organic Frameworks for Hyperuricemia Treatment

Zeyu Li,‡ Liecong Xue,‡ Junxian Yang, Stefan Wuttke,Peiying He, Chuanyi Lei, Haowei Yang, Liang Zhou, Jiangfan Cao, Anna Sinelshchikova, Guansheng Zheng, Jimin Guo,Jiangguo Lin, Qi Lei, C. Jeffrey Brinker, Kaisheng Liu,* and Wei Zhu**

Z. Li, L. Xue, P. He, C. Lei, L. Zhou, J. Cao, G. Zheng, and Prof. W. Zhu
MOE International Joint Research Laboratory on Synthetic Biology and Medicines, School of Biology and Biological Engineering, South China University of Technology, Guangzhou, 510006, P. R. China
E-mail: zhuwei86@scut.edu.cn

J. Yang and Prof. J. Lin
Medical Research Institute, Guangdong Provincial People's Hospital (Guangdong Academy of Medical Sciences), Southern Medical University, Guangzhou, 510000, P. R. China

Prof. S. Wuttke
BCMaterials, Basque Center for Materials, UPV/EHU Science Park, 48940 Leioa, Spain
IKERBASQUE, Basque Foundation for Science, 48009 Bilbao, Spain
E-mail: stefan.wuttke@bcmaterials.net

Haowei Yang
China National Tobacco Corporation, No.55 South Yuetan Boulevard Xicheng District, Beijing 100045, P. R. China

Dr. Anna Sinelshchikova
BCMaterials, Basque Center for Materials, UPV/EHU Science Park, 48940 Leioa, Spain

Prof. J. Guo
College of Materials Sciences and Engineering, Beijing University of Chemical Technology, Beijing, 100029, P. R. China

Prof. Q. Lei

The Second Affiliated Hospital, State Key Laboratory of Respiratory Disease, Guangdong Provincial Key Laboratory of Allergy and Clinical Immunology, Guangzhou Medical University, Guangzhou, 510260, P.R. China

Prof. C. J. Brinker
Center for Micro-Engineered Materials and the Department of Chemical and Biological Engineering, The University of New Mexico, Albuquerque, New Mexico 87131, USA

Prof. K. Liu
Shenzhen People's Hospital (The Second Clinical Medical College, Jinan University; The First Affiliated Hospital, Southern University of Science and Technology), Shenzhen 518020, P. R. China
E-mail: liukaisheng@szhospital.com

Table of Contents

Section S1. General information 4
Section S2. Nanoparticles synthesis 5
Section S3. Enzyme activity test 5
Section S4. Determination of enzyme concentrations 6
Section S5. Fluorescent labeling of enzymes 6
Section S6. Enzyme stability test of UCZ 6
Section S7. Synthesis of red blood cell superstructures 7
Section S8. Hemolysis assay 7
Section S9. Chemiluminescence 7
Section S10. Capability of reversibly binding oxygen 8
Section S11. Characterization of RBC membrane proteins 8
Section S12. Cell viability assay 8
Section S13. Detection of reactive oxygen species (ROS) 9
Section S14. In vivo studies of uric acid degradation 9
Section S15. Supplementary figures 11
Section S16. Supplementary references 36

Section S1. General information

Reagents. All chemicals and reagents were used as received. Uricase (UOX, 10 units/mg) was purchased from Sigma-Aldrich (Shanghai, China). Zinc nitrate hexahydrate ($Zn(NO_3)_2 \cdot 6H_2O$), 2-methylimidazole (2-MIM), and Cy7-NHS were from Aladdin (Shanghai, China). Uric acid, glutaraldehyde, fluorescein isothiocyanate, hypoxanthine, potassium oxonate, tannic acid (TA), and ABTS diammonium salt (2,20-azino-bis-3-ethylbenzthiazoline-6-sulphonic acid, ABTS) were purchased from Macklin (Shanghai, China). Catalase (CAT, 40,000 units/mg) was obtained from Solarbio (Beijing, China). 3-[4,5-dimethylthiazol-2-yl-]-2,5-diphenyltetrazolium-bromide (MTT) and 2',7'-Dichlorofluorescin diacetate (DCFH-DA) were provided by the Beyotime Institute of Biotechnology (Shanghai, China). Phosphate buffered solutions (PBS) were obtained from Tansoole (Shanghai, China). Sodium chloride (NaCl) was purchased from General-Reagent (Shanghai, China). Hydrogen peroxide (H_2O_2, 30%) was purchased from Chengdu Cologne Chemicals Co., Ltd. (Chengdu, China). Dulbecco's modified of Eagle's medium (DMEM), fetal bovine serum (FBS), and penicillin streptomycin were purchased from Gibco (Logan, USA). Ultrapure water (18.2 MΩ; Millipore Co., USA) was used to prepare all buffers and was used throughout all experiments.

Characterization methods. Scanning electron microscopy (SEM) analyses and energy-dispersive X-ray spectroscopy (EDS) elemental mappings were performed on a field-emission scanning electron microscope (Merlin, Zeiss, Germany). Transmission electron microscopy (TEM) and high-resolution TEM (HRTEM) imaging were carried out using a Talos L120c transmission electron microscope (Thermo Fisher Scientific, USA) at 120 kV. The X-ray diffraction (XRD) was measured by an X-ray diffractometer (Rigaku Miniflex, Japan). Nitrogen adsorption-desorption isotherms and the pore size distribution were determined at 77 K by N_2 adsorption-desorption measurement (BSD-PM1, 3 BSD instruments, China). The UV-Vis absorption measurements were recorded on a UV-Vis spectrophotometer (UV-2600, Shimadzu, Japan). Fluorescence emission measurements were carried out using a fluorescence spectrometer (Shimadzu RF-6000). Fourier transform infrared spectroscopy (FT-IR) spectra were measured by an FT-IR spectrometer (Spectrum Two, PerkinElmer, USA). Fluorescent images were obtained

using the LAS X software on a DMi8 microscope (Leica, Germany) operated in channel mode. AFM was used to observe the morphology of RBC in contact mode, the probe was triangular silicon nitride cantilever (OXFORD instruments, PNP-TR-Au) with a nominal spring constant of 0.08 N/m, and the morphological images were analyzed and processed with the software of AFM instrument AR 16.25.226.

Section S2. Nanoparticles synthesis

ZIF-8 NPs synthesis. ZIF-8 NPs were synthesized according to the reported approach with slight modifications[1]. Prepare a solution of 229.6 mg/mL of 2-MIM and 11.9 mg/mL of $Zn(NO_3)_2·6H_2O$. 2 mL of 2-MIM was mixed with 2 mL of $Zn(NO_3)_2·6H_2O$, stirred at room temperature for 15 min, and centrifuged at 8000 rpm for 10 min to obtain a white precipitate, which was washed several times with deionized water.

UCZ synthesis. Based on the previous method, UOX, CAT, and 2-MIM solutions were initially mixed, followed by the addition of $Zn(NO_3)_2·6H_2O$ solution, and the mixture was agitated at room temperature for 15 min. A white precipitate was produced and washed multiple times with deionized water. UCZ with different ratios of UOX and CAT were prepared by the same procedure.

Section S3. Enzyme activity test

Degradation kinetics of UA. In brief, 2 mg UA was dissolved in 2 mL borate buffer (pH 8.5, 100 mM) and sonicated until completely dissolved. Then, the UA working solution was formed by diluting the above solution 20 times with borate buffer. The enzyme solution to be tested was added with UA working solution, respectively. The reaction system was incubated at 37 ºC, and then the absorbance of the mixtures at 290 nm was determined using UV-Vis.

Kinetic studies of H_2O_2 generation. The kinetics of H_2O_2 generation and elimination were monitored according to the following procedure. Firstly, an ABTS solution (1.0 mg/mL) was mixed with HRP to obtain a substrate solution containing 0.4 mM ABTS and 0.005 U/mL HRP. 100 μL of the substrate solution was injected into a 96-well plate.

Meanwhile, 20 μL of reaction solution of UA with UOX or UCZ was taken at a fixed time and transferred to the aforesaid 96-well plate. The absorbance change at 415 nm was recorded by a microplate reader.

Section S4. Determination of enzyme concentrations

Enzyme concentrations were determined using a BCA assay kit. Briefly, BCA working solution (BWS) was prepared by mixing 50 volumes of Reagent A and 1 volume of Reagent B and then establishing a standard curve (0.03125, 0.0625, 0.125, 0.25, 0.5, 0.75, and 1 mg/mL) with the corresponding enzymes. At the same time, 25μL of enzyme was added into the 200 μL of BWS and incubated at 37 ºC for 30 min. The absorbance at 562 nm of each sample was read out using BioTek (Agilent Technologies, USA).

Section S5. Fluorescent labeling of enzymes

Synthesis of Cy7 labeled UOX. Firstly, 50 μL of Cy7 in DMSO (10 mg/mL) was gradually added to 1 mL enzyme solution (10 mg/mL UOX containing 50 mM Boric acid buffer, pH 8.5). This reaction was carried out overnight at 4 ºC. The labeled proteins were then dialyzed with Boric acid buffer (50 mM, pH = 8.5) to remove free Cy7 and stored at 4 ºC for further use.

Synthesis of FITC labeled CAT. First, 50 μL of FITC in DMSO (10 mg/mL) was gradually added to 1 mL enzyme solution (10 mg/mL CAT). This reaction was conducted overnight at 4 ºC. Then the labeled proteins were dialyzed to remove free FITC and stored at 4 ºC for further use.

Section S6. Enzyme stability test of UCZ

The effects of temperature, pH, and trypsin digestion on the activity of UOX were explored. The maximum activity of free UOX and UCZ was taken as 100%, respectively. The residual activity was defined as the percentage of the maximum activity. For the temperature stability test, UOX and UCZ were mixed with UA (200 μM), respectively. Then these mixtures were incubated at different temperatures (30, 40, 50, 60, and 70 °C) for 1 h, and the absorbance changes at 290 nm were measured to evaluate the residual

activities of different samples. Similarly, for the pH stability test, UOX and UCZ were placed in PBS at different pH values (5, 6, 7, 8, 9, 10, and 11) and incubated for 1 h at 37 °C. For the trypsin digestion test, UOX and UCZ were incubated with 1 mg/mL trypsin at 37 °C, and samples were taken at different times (0, 10, 20, 40, and 60 min) to determine the residual catalytic activities.

Section S7. Synthesis of red blood cell superstructures

500 μL 1X PBS (pH 5) solution containing 400 μg/mL UCZ NPs was used to suspend 5 million RBCs. After 10 s swirling and 20 s incubation, 500 μL of 32 μg/mL tannic acid in 1X PBS (pH 7.4) solution were added with 30 s vigorous mixing. After forming the red blood cell superstructures, 1X PBS (pH 7.4) was used to rinse them off and store them.

Section S8. Hemolysis assay

Native RBCs and UCZR were washed with 1X PBS (pH 7.4) solution before being suspended in 1X PBS (pH 7.4) solution at room temperature for 7 days. Water and 1X PBS (pH 7.4) solution were employed as positive controls (100% hemolysis) and negative controls (0% hemolysis). The absorbance of hemoglobin in the supernatant was measured by a BioTek microplate reader (Synergy HTX) at 540 nm to calculate the hemolysis percentage following centrifugation (200 g, 5 min). The hemolysis percentage of each sample was determined using the reported equation. Percent hemolysis (%) = 100×(sample abs_{540nm}-negative control abs_{540nm})/(positive control abs_{540nm}-negative control abs_{540nm})

Section S9. Chemiluminescence

The oxygen carrying capacity of RBCs was assessed using Luminol-based chemiluminescence[2]. Briefly, 5 mL of water was sonicated to dissolve 35 mg sodium perborate, 250 mg sodium carbonate, and 100 mg luminol. The luminol solution was left in the dark for 5 min without being touched. For imaging, 1 mL of luminol solution was added to 4 mL samples (20 million native RBCs or RBC superstructures) in 1X PBS (pH 7.4) solution.

Section S10. Capability of reversibly binding oxygen

By examining variations of UV–Vis absorption spectra (300–700 nm) in oxygenated and deoxygenated solutions, the reversible binding ability of oxygen was discovered. Nitrogen gas was blasted into the sample solution to remove the majority of the oxygen for complete deoxygenation. Sodium dithionite ($Na_2S_2O_4$) was added after 2 h, and a BioTek microplate was used to scan UV–Vis absorption spectrum. During oxygenation, the sample solution was exposed to atmospheric oxygen for more than 2 h, and UV–Vis absorption spectrum was captured. Repeat the process three times.

Section S11. Characterization of RBC membrane proteins

Erythrocyte ghosts were prepared by incubating the RBCs or UCZR in 0.25X PBS overnight and collecting by centrifugation (12,000 g, 10 min), and then the membrane proteins were extracted with the Membrane Protein Extraction Kit (Sangon). The proteins were analyzed by sodium dodecyl sulfate-polyacrylamide gel electrophoresis (SDS-PAGE). Briefly, the RBC membrane proteins were prepared in SDS sample buffer and heated at 95 °C for 5 min. All the obtained proteins were dissolved in 5X Protein Loading Dye (Sangon) and then run on 8% SDS-polyacrylamide gel at 120 V for 1.5 h. The polyacrylamide gel was then stained with Coomassie Brilliant Blue R250 Protein Stain Reagent for 0.5 h and washed overnight to visualize.

Section S12. Cell viability assay

We carried out cell culture according to standard procedures. HSF cells were cultured in Dulbecco's modified Eagle's medium (DMEM) media containing 10% FBS and 1% antibiotics with 5% CO_2 at 37 °C. Cells were passaged at approximately 70% confluency. The *in vitro* cytotoxicities of UCZ and UCZR were determined by MTT assays. Briefly, 100 μL of cells (10^4 cells/mL) were seeded into the 96-well plate and cultivated for 24 h at 37 °C in DMEM. The cells were then cultured with 100 μL of various concentrations of UCZ NPs or UCZR solutions. After 12 h of incubation, 20 μL MTT (5 mg/mL) was added into each well and further incubated for 4 h. Then the supernatant was replaced by 150 μL DMSO to dissolve the formazan. After the complete dissolution of formazan, the luminescence readings were recorded by a BioTek microplate reader at 570 nm. The

percent cell viability was calculated relative to the untreated control cells. The viability of 4T1 cells in the presence of uric acid oxidation reaction catalyzed by UOX, UCZ, and UCZR was tested using a similar procedure. The native UOX, UCZ, or UCZR (20 mU/well), were first incubated with the cells (10^4 cells) for 1 h. Then equal amount of saturated uric acid working solutions (10 μL) were added into each well and incubated with cells for another 12 h at 37 °C.

Section S13. Detection of reactive oxygen species (ROS)

The reactive oxygen species detection kit utilizes a fluorescent probe 2',7'-dichlorofluorescein diacetate (DCFH-DA) to detect reactive oxygen species. DCFH-DA itself has no fluorescence, and can freely permeate the cell membrane. Once inside the cell, it is hydrolyzed by intracellular esterase to produce DCFH. Intracellular reactive oxygen species can oxidize DCFH to generate DCF with fluorescence. 4T1 cells inoculated in confocal culture dishes were treated with various materials. Subsequently, the original medium was replaced with a fresh culture medium containing UOX, UCZ, or UCZR mixed with UA. After 12 h, the intracellular ROS levels were monitored by DCFH-DA staining and observed under a fluorescence microscope.

Section S14. *In vivo* studies of uric acid degradation

Male KM mice (6 weeks old) were purchased from Hunan Slyke Jingda Experimental Animal Co., Ltd. Animal experiments were conducted with reference to animal welfare. All animal experiments were carried out in the SPF Laboratory of the Animal Experiment Center, South China University of Technology. All animal experiments were approved by the Institutional Animal Care and Use Committee of South China University of Technology (Approval NO.2021011). Before the formal experiment, the animals were fed adaptively for one week, during which they were free to eat and drink. All mice were raised at a controlled temperature (24 °C) and relative humidity (50%) on a 12-h light/dark cycle. The suspension was prepared with 0.5% sodium carboxymethyl cellulose (CMC-Na) as the solvent. Blank group, 100 mg/kg potassium oxonate + 200 mg/kg hypoxanthine model group, free UOX group, UCZ group, and UCZR group were established. Briefly, the mice were perfused with hypoxanthine (200 mg/kg) by

intraperitoneal administration, while potassium oxonate (100 mg/kg) was performed to mice by subcutaneous administration. After 1 h of modeling, all drugs were injected into mice via vein tail at a dosage of 25 U/kg (UOX) body weight. Basal uric acid levels in the blood of mice were measured before injection. Blood samples were collected from the tail vein of mice at each time points (2 h, 4 h, and 6 h) and centrifuged at 3500 r/min for 10 min to separate the plasma. The samples were frozen at -20 °C for subsequent analysis, and uric acid levels were assessed using a blood biochemical instrument. For LDH, ALT, AST, BUN, and CRE measurement, blood samples were collected from the orbital venous plexus of mice with blood collection needle. Then the blood samples were stored overnight at 4 °C in a coagulant tube, which allows the blood to coagulate naturally. Afterward, samples were centrifuged at 2000 rpm for 10 minutes, and the supernatant was collected for blood biochemical analyzer on EMO-EXPRES. For WBC measurement, the routine blood tests were completed within 4 h with a BC-5000 vet. The contents of interleukin-6 (IL-6) and tumor necrosis factor (TNF-α) in serum were assessed by mouse ELISA kits, respectively, following the provided protocols. For H&E staining analysis, the mice were euthanized 24 h after the treatment, and the main organs (heart, liver, spleen, lungs, and kidney) were taken for H&E staining analysis.

Section S15. Supplementary figures

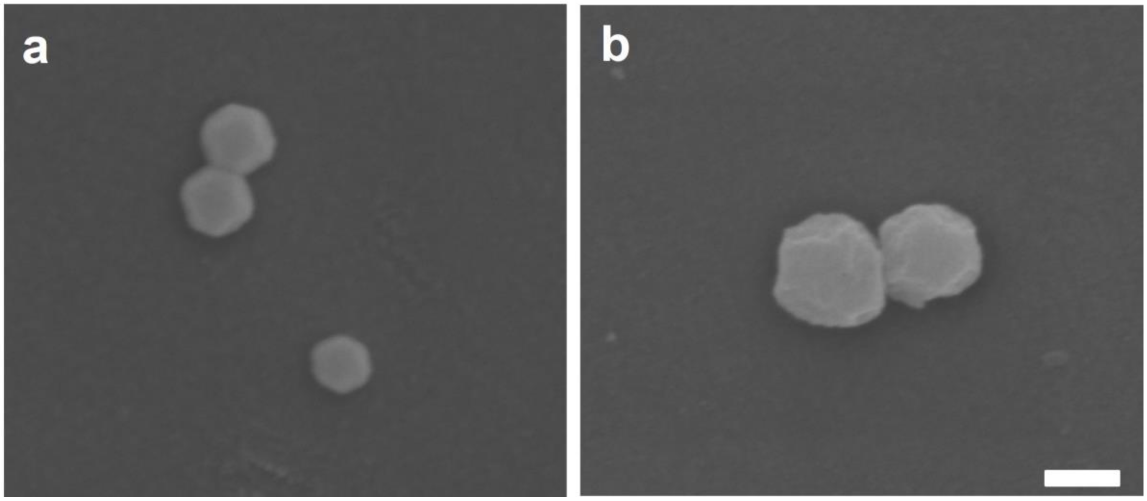

Figure S1. SEM images of a) ZIF-8 nanoparticles and b) UCZ. Scale bar, 200 nm.

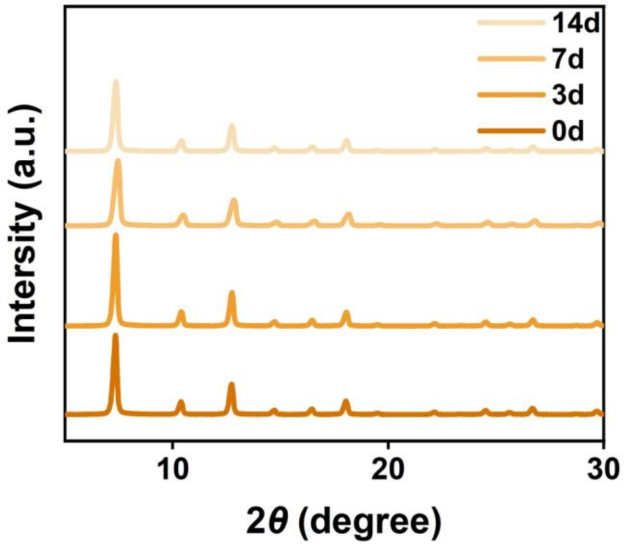

Figure S2. XRD patterns of UCZ stored at different times.

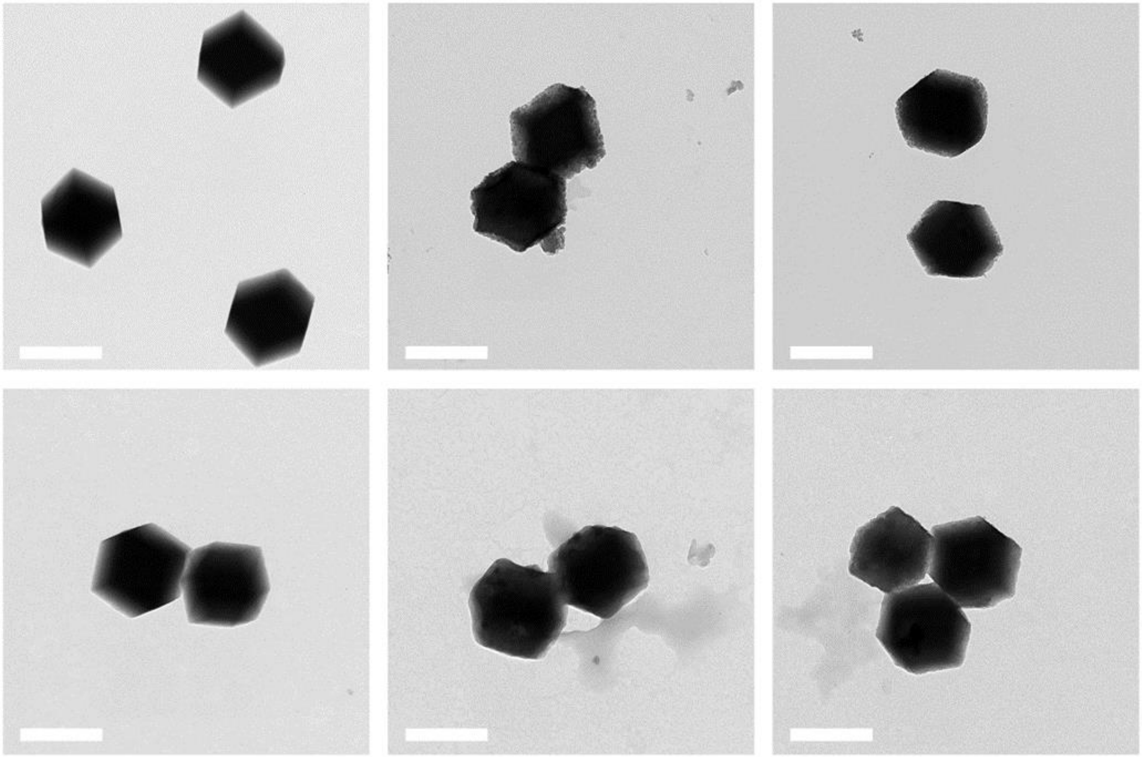

Figure S3. TEM images of ZIF-8 nanoparticles stored in PBS (above) and plasma (below) for 0 days, 3 days, and 7 days (from left to right). Scale bar: 500 nm.

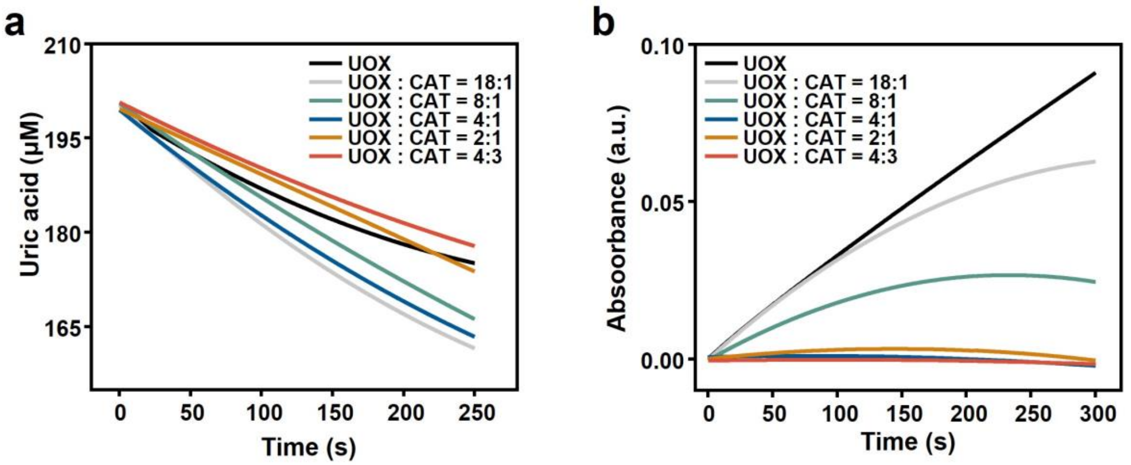

Figure S4. a) UA degradation under different ratios of UOX and CAT. b) H_2O_2 generation under different ratios of UOX and CAT.

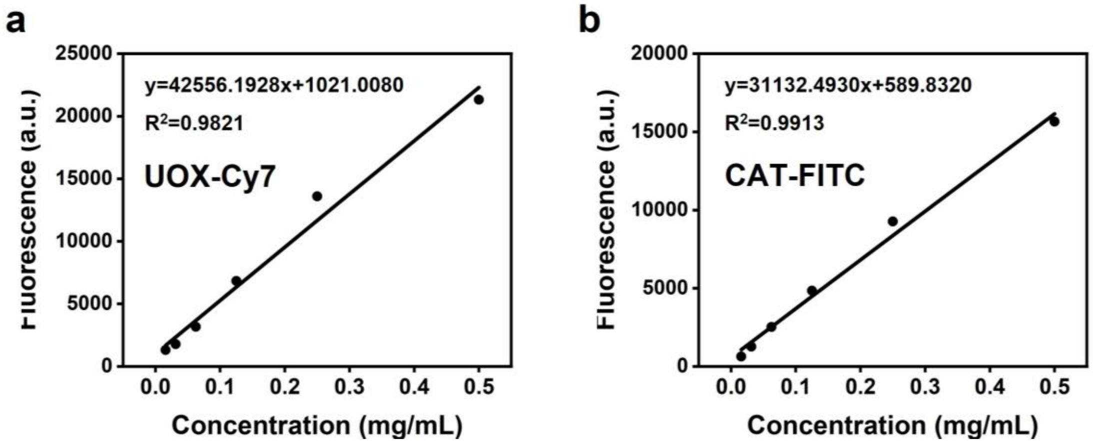

Figure S5. Standard curve represents the relationship between absorbance and enzyme concentration. a) UOX-Cy7 and b) CAT-FITC.

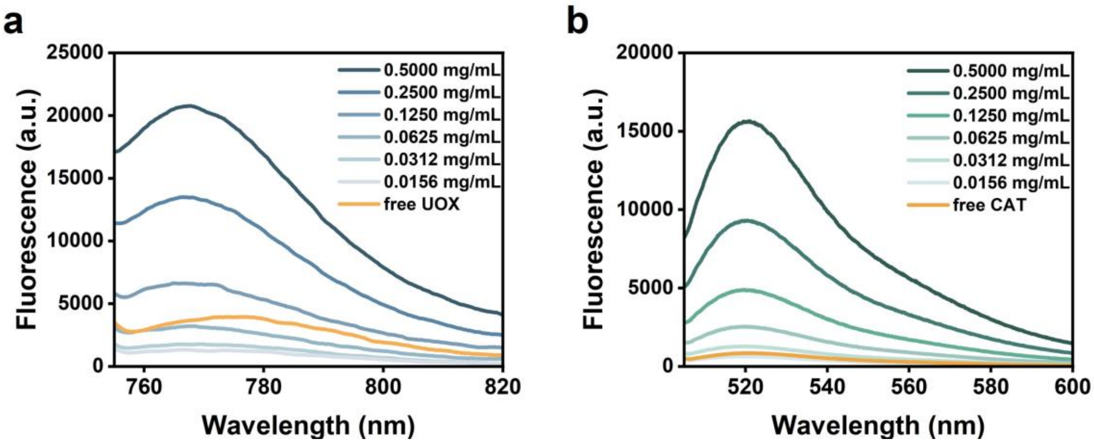

Figure S6. Fluorescence intensity of supernatant after reaction. a) UOX-Cy7 and b) CAT-FITC.

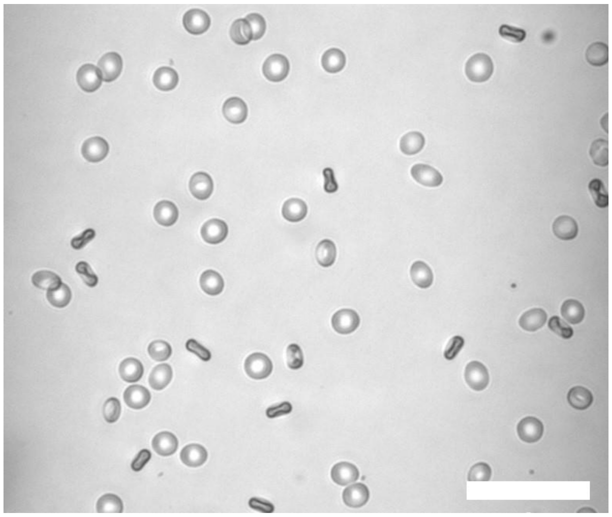

Figure S7. Bright field image of RBCs. Scale bar, 100 μm.

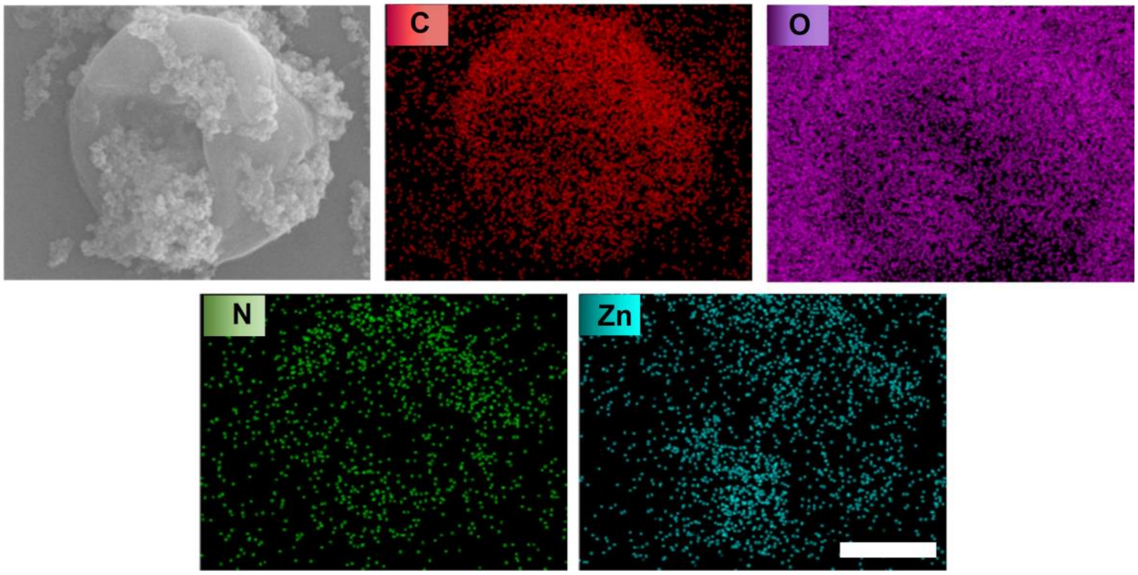

Figure S8. SEM mapping (Zn, C, O, and N) of UCZR. Scale bar, 2 μm.

.

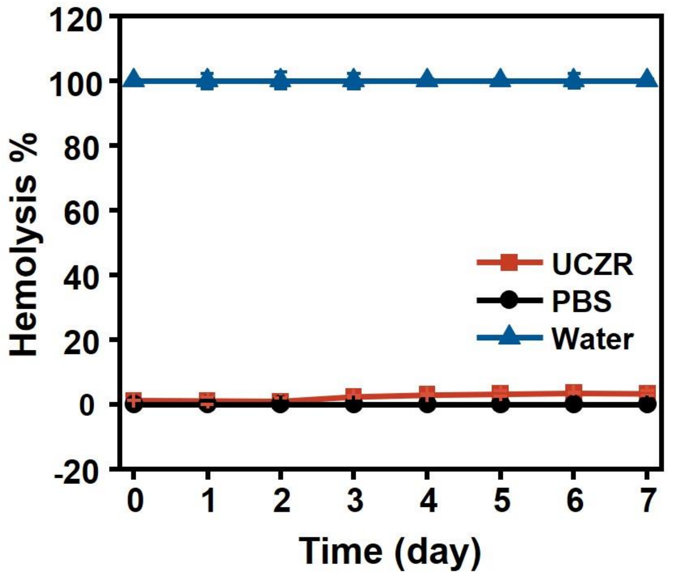

Figure S9. Time-dependent hemolysis rate of UCZR in 1X PBS.

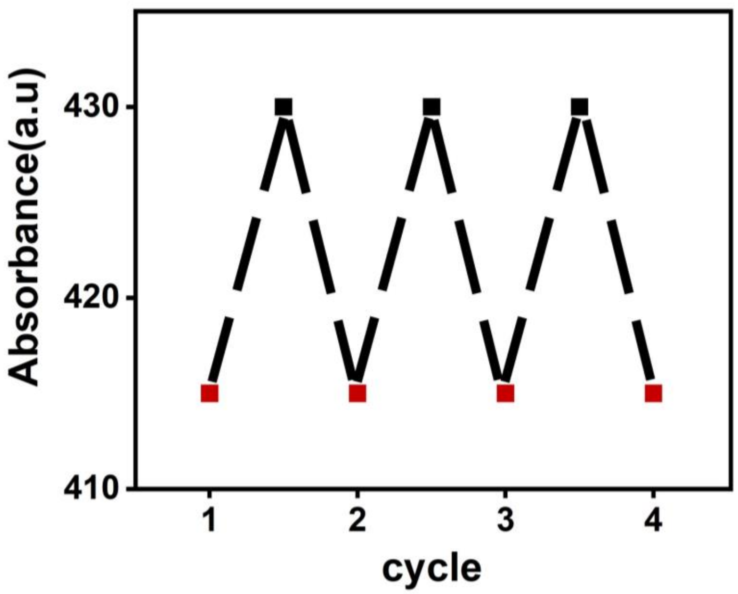

Figure S10. The reversible transfer between the oxygenated and deoxygenated states of UCZR.

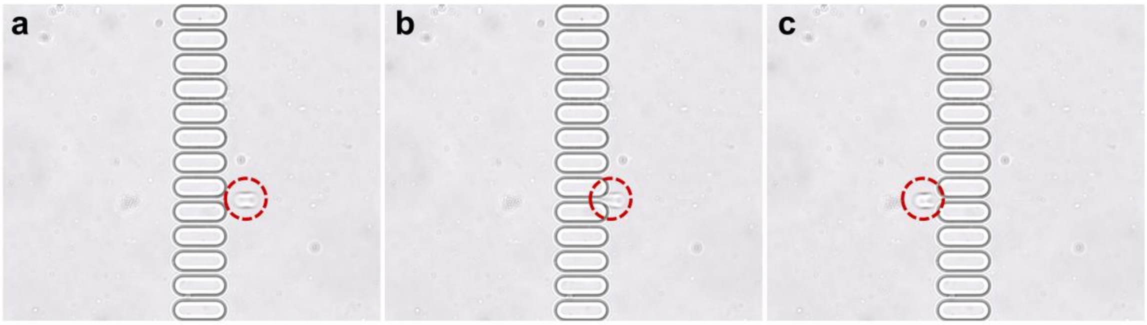

Figure S11. The pictures of ZIF-8-RBC biohybrids via microfluidic chip channels. a) ZIF-8-RBC biohybrids before passage, b) ZIF-8-RBC biohybrids during passage, and c) ZIF-8-RBC biohybrids after passage.

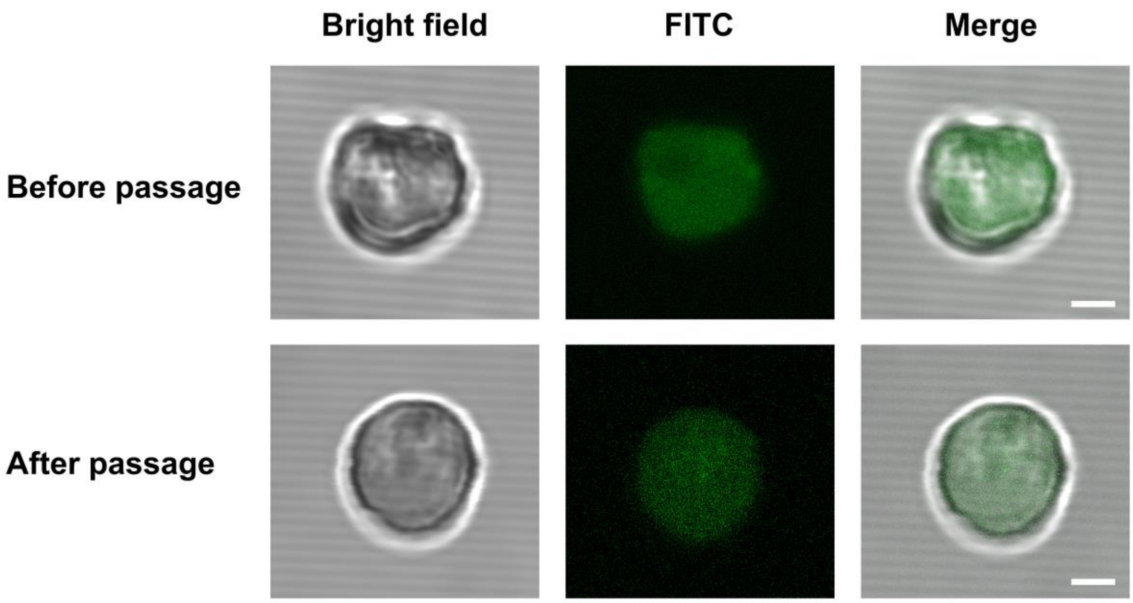

Figure S12. Representative images of ZIF-8-RBC biohybrids before and after passing through the chip channels (bright field, FITC, merged image, from left to right). Scale bars: 2 μm.

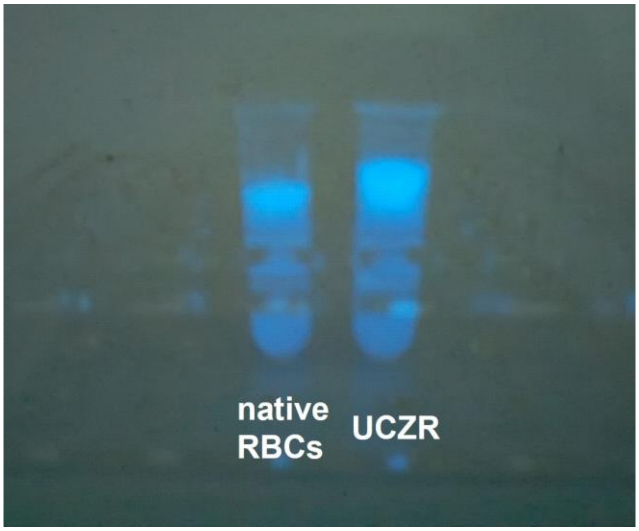

Figure S13. Luminescence picture of RBCs and UCZR.

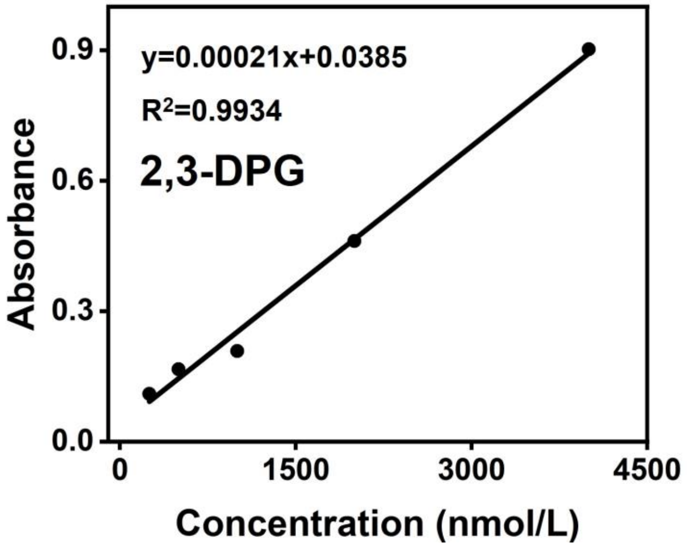

Figure S14. The standard curve of 2,3-DPG.

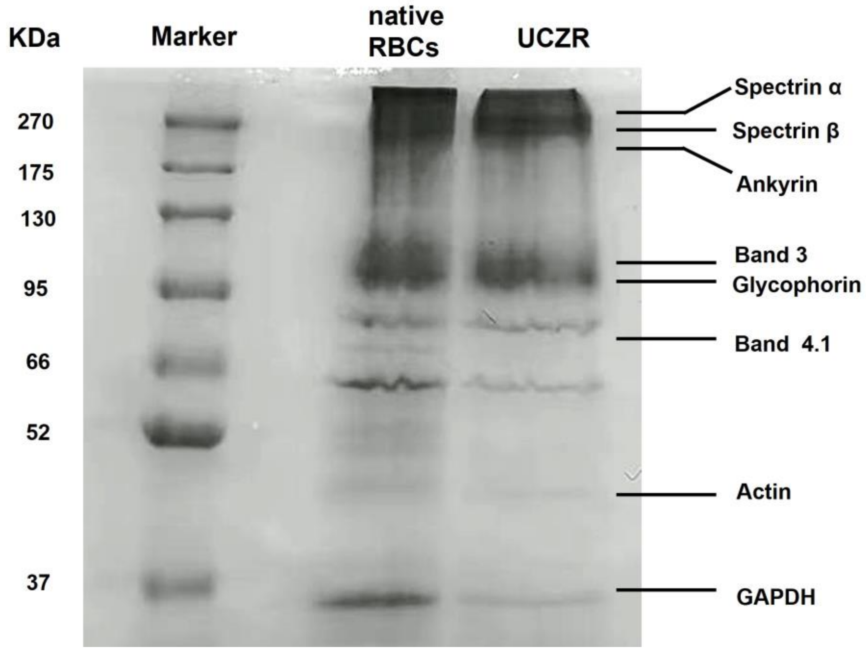

Figure S15. SDS-PAGE electrophoresis of erythrocyte membrane protein.

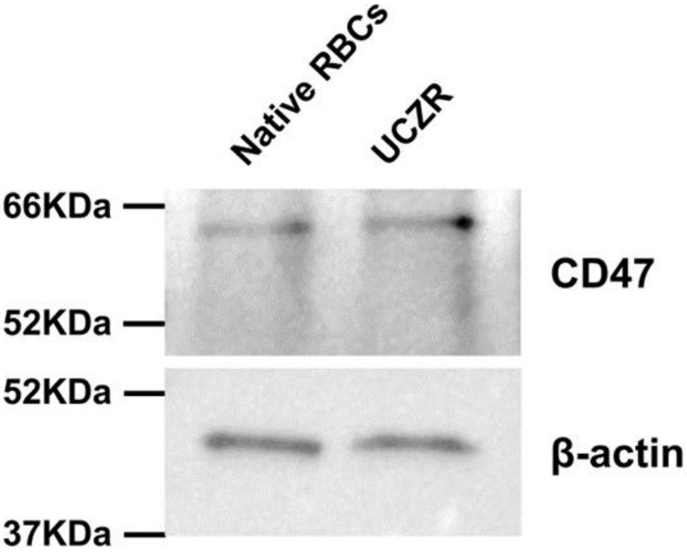

Figure S16. Western blotting analysis of Native RBC membrane, UCZR membrane for characteristic CD47 of the membrane marker.

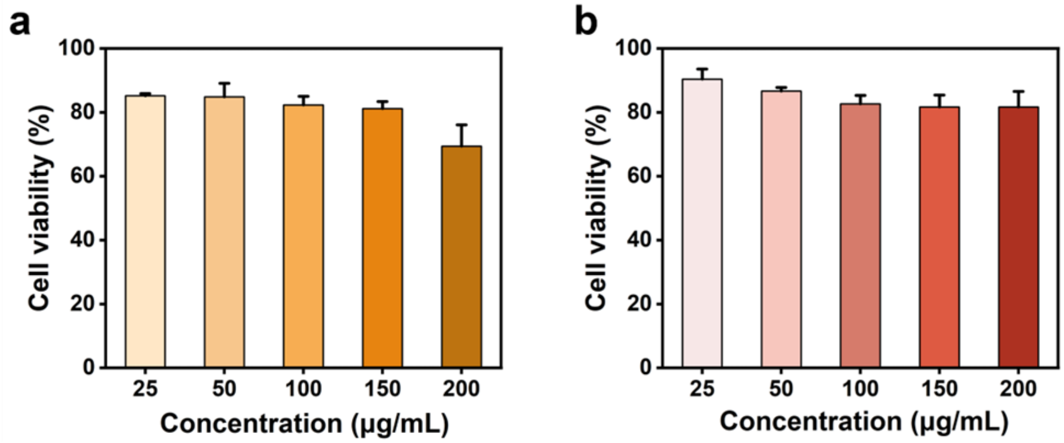

Figure S17. a) Cytotoxicity of nanoparticles of UCZ at different concentrations. b) Cytotoxicity of UCZR at different UCZ concentrations. (mean± standard deviation, n = 5).

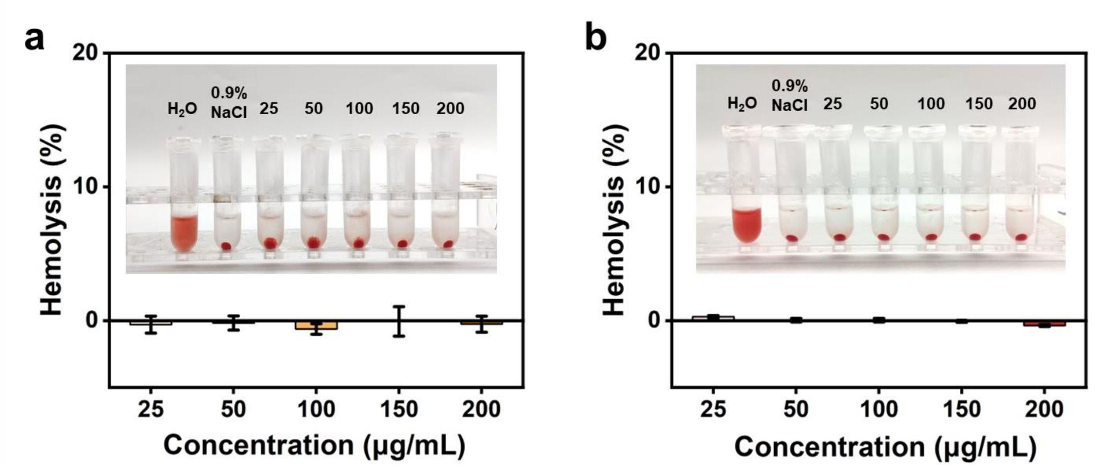

Figure S18. a) Hemolysis rate test of different concentrations of UCZ. b) Hemolysis rate test of different UCZ concentrations of UCZR. (mean± standard deviation, n = 3).

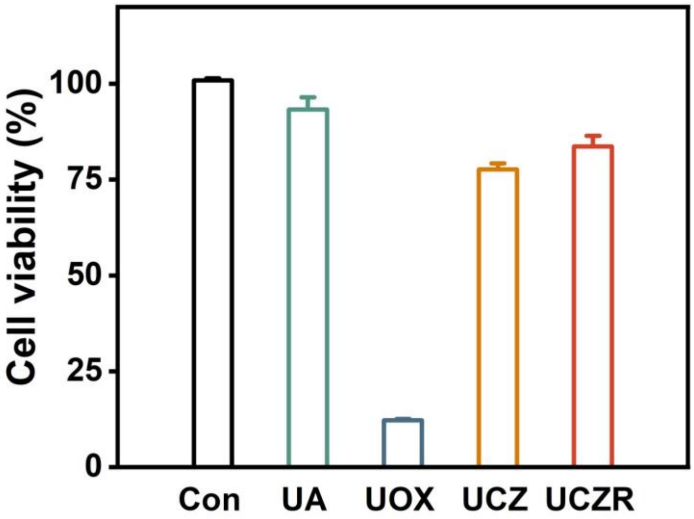

Figure S19. Cell viability assays after treated with UOX, UCZ, and UCZR in the presence of UA.

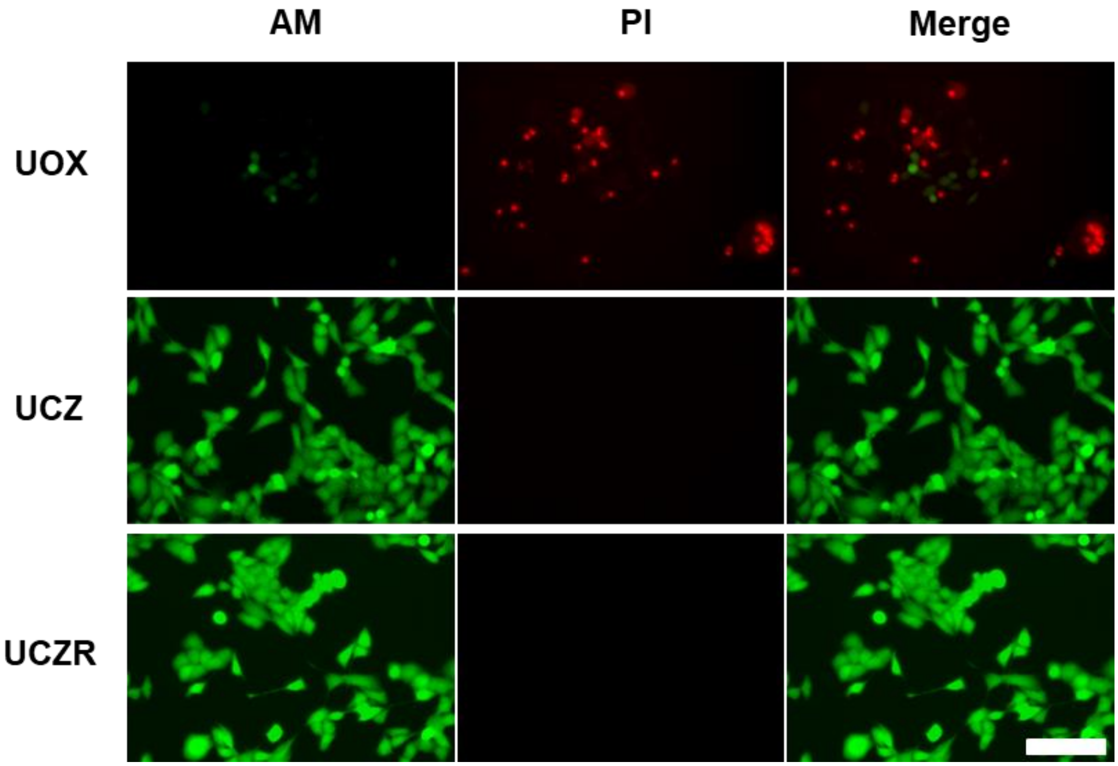

Figure S20. Calcein-AM/PI double staining of 4T1 cells treated with UOX, UCZ, and UCZR in the presence of UA. Scale bar, 2 μm.

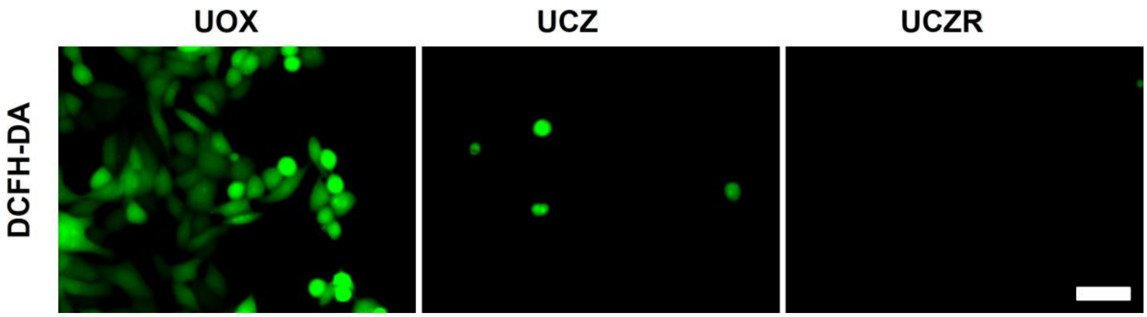

Figure S21. The ROS level in UOX, UCZ, and UCZR treatments in the presence of UA. Scale bar, 50 μm.

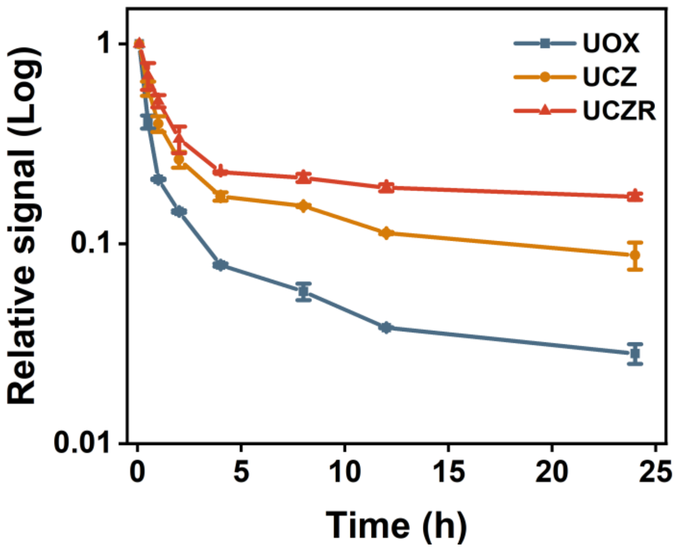

Figure S22. Semilog plot of the circulation time of UOX, UCZ, and UCZR.

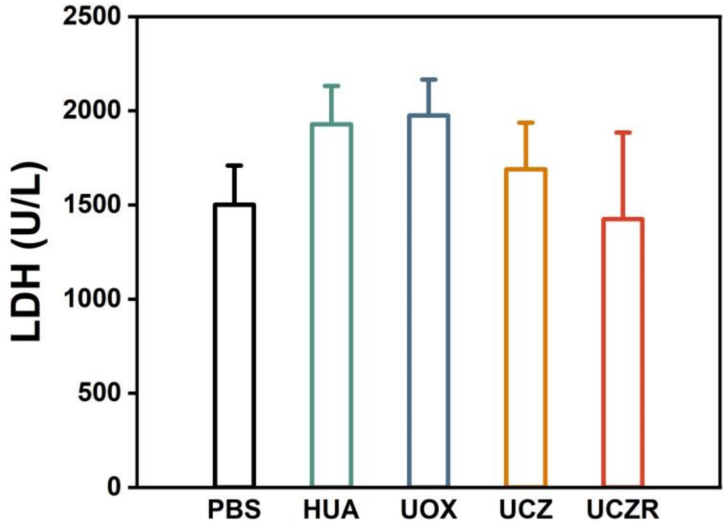

Figure S23. LDH levels of mice in different groups (mean± standard deviation, n = 3, two-tailed t-test).

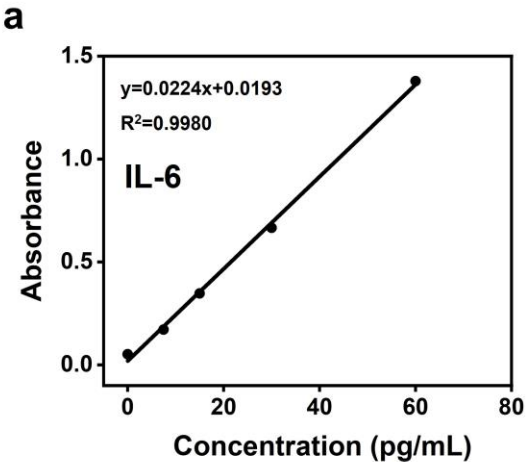

Figure S24. Standard curves of inflammatory cytokines. a) IL-6， b) TNF-α.

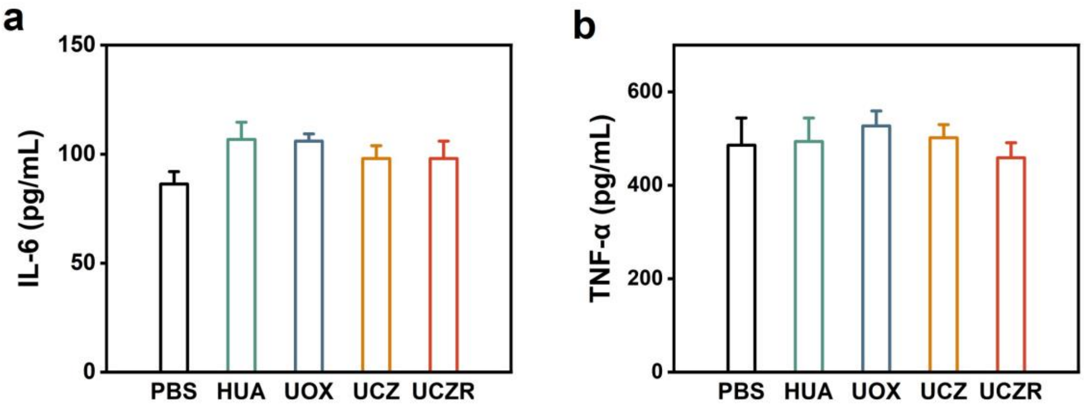

Figure S25. Inflammatory factor levels of mice with different samples. a) IL-6 and b) TNF-α (mean± standard deviation, n = 3, two-tailed t-test).

Section S16. Supplementary references

[1] Y. Pan, Y. Liu, G. Zeng, L. Zhao, Z. Lai, *Chemical Communications* **2011**, 47, 2071.

[2] N. Doshi, A. S. Zahr, S. Bhaskar, J. Lahann, S. Mitragotri, *Proceedings of the National Academy of Sciences* **2009**, 106, 21495.
